# A Targeting Microbubble for Ultrasound Molecular Imaging

**DOI:** 10.1371/journal.pone.0129681

**Published:** 2015-07-10

**Authors:** James Shue-Min Yeh, Charles A. Sennoga, Ellen McConnell, Robert Eckersley, Meng-Xing Tang, Sussan Nourshargh, John M. Seddon, Dorian O. Haskard, Petros Nihoyannopoulos

**Affiliations:** 1 National Heart and Lung Institute, Imperial College London, London, United Kingdom; 2 Department of Cardiology, Hammersmith Hospital, London, United Kingdom; 3 Imaging Sciences Department, Medical Research Council, Imperial College London, London, United Kingdom; 4 Department of Chemistry, Imperial College London, London, United Kingdom; 5 Department of Bioengineering, Imperial College London, London, United Kingdom; 6 William Harvey Research Institute, Queen Mary, University of London, London, United Kingdom; Baker IDI Heart and Diabetes Institute, AUSTRALIA

## Abstract

**Rationale:**

Microbubbles conjugated with targeting ligands are used as contrast agents for ultrasound molecular imaging. However, they often contain immunogenic (strept)avidin, which impedes application in humans. Although targeting bubbles not employing the biotin-(strept)avidin conjugation chemistry have been explored, only a few reached the stage of ultrasound imaging *in vivo*, none were reported/evaluated to show all three of the following properties desired for clinical applications: (i) low degree of non-specific bubble retention in more than one non-reticuloendothelial tissue; (ii) effective for real-time imaging; and (iii) effective for acoustic quantification of molecular targets to a high degree of quantification. Furthermore, disclosures of the compositions and methodologies enabling reproduction of the bubbles are often withheld.

**Objective:**

To develop and evaluate a targeting microbubble based on maleimide-thiol conjugation chemistry for ultrasound molecular imaging.

**Methods and Results:**

Microbubbles with a previously unreported generic (non-targeting components) composition were grafted with anti-E-selectin F(ab’)_2_ using maleimide-thiol conjugation, to produce E-selectin targeting microbubbles. The resulting targeting bubbles showed high specificity to E-selectin *in vitro* and *in vivo*. Non-specific bubble retention was minimal in at least three non-reticuloendothelial tissues with inflammation (mouse heart, kidneys, cremaster). The bubbles were effective for real-time ultrasound imaging of E-selectin expression in the inflamed mouse heart and kidneys, using a clinical ultrasound scanner. The acoustic signal intensity of the targeted bubbles retained in the heart correlated strongly with the level of E-selectin expression (|r|≥0.8), demonstrating a high degree of non-invasive molecular quantification.

**Conclusions:**

Targeting microbubbles for ultrasound molecular imaging, based on maleimide-thiol conjugation chemistry and the generic composition described, may possess properties (i)–(iii) desired for clinical applications.

## Introduction

Ultrasound molecular imaging has been achieved using targeting microbubbles which contain targeting ligands on the bubble-shell [1]. Following *intravenous* (*iv*) administration, these echogenic bubbles circulate and accumulate in regions expressing the molecules targeted, depicted on ultrasound pictures as areas of bright signals locating the molecules of interest. This technique has allowed molecular imaging of pathophysiological processes such as inflammation, angiogenesis and thrombosis, and has potential for clinical applications (Table A in S1 File).

However, targeting bubbles often employed biotin-(strept)avidin conjugation chemistry for grafting targeting ligands to the bubble-shell (Table A in S1 File), which is best avoided for use in humans because of the high immunogenicity of (strept)avidin. Targeting bubbles not employing the biotin-(strept)avidin conjugation chemistry have been developed, these included those based on maleimide-thiol or other conjugation chemistries (reviewed in Yeh 2010) [2]. Only about half of them were tested in animals for ultrasound molecular imaging (Table A in S1 File), none were reported/evaluated to show all three of the following properties desired for clinical applications: (i) low degree of non-specific bubble retention in more than one non-reticuloendothelial tissue (allowing specific molecular detection in different tissues); (ii) effective for real-time imaging (allowing ready visualization of the molecular target(s) by the bedside, and better assessment of moving objects such as the beating heart); and (iii) effective for quantification of molecular targets to a high degree of quantification (increasing diagnostic power). Furthermore, enabling disclosure of the compositions and methodologies for reproducing these bubbles are often withheld (Table A in S1 File).

In this study, we developed and disclosed the full methodology for producing a targeting microbubble, based on maleimide-thiol conjugation chemistry and a previously unreported generic (non-targeting components) composition. E-selectin (Esel), an adhesion molecule expressed on the endothelium in inflammation, was chosen as a prototype molecule for bubble targeting. We showed that the Esel targeting bubble developed was effective for molecular imaging in inflammation of the heart and kidneys *in vivo*. The bubble possessed all three properties (i)-(iii). Based on the maleimide-thiol conjugation chemistry and properties (i)-(iii), the bubble is a potentially candidate for further development towards clinical translation.

## Materials and Methods

### Antibodies

MES-1 monoclonal antibody (mAb), a rat IgG2a,κ against mouse Esel, and its F(ab’)_2_ fragment were provided by Dr D Brown (UCB-Celltech, UK) [3]. MES-1 labelled with 7 Alexa Fluor 488 fluorescence dye (AF488-MES-1) and reduced MES-1 F(ab’)_2_ containing 2 thiol groups per F(ab’)_2_ from tris(2-carboxyethyl)phosphine hydrochloride reduction was prepared as described in the S1 File. MEC13.3 mAb, a rat IgG2a,κ against mouse PECAM-1 (BD Biosciences), allophycocyanin-labelled mAb against mouse PECAM-1 (BD Pharmingen), rat IgG2a,κ isotype negative control mAb (BD Biosciences) and biotinylated rabbit mAb against rat IgG2a (Vector Laboratories) were purchased.

### Animals

Wild-type (WT) mice were adult male C57BL/6J (Charles River, UK). Esel knock-out (KO) mice were adult male Esel homozygote KO on C57BL/6J background [4], bred locally from mice donated by Dr K Norman and Prof P Hellewell (University of Sheffield, UK). All animal work was carried out under licences granted by the UK Home Office under the Animals (Scientific Procedures) Act 1986. All animals were sacrificed humanely by dislocation of the neck, or overdose of anesthetic followed by dislocation of the neck. Ethical approval was obtained from Imperial College London's Ethical Review Panel.

### Mouse Model of Lipopolysaccharide-Induced Systemic Inflammation (Experimental Endotoxemia)

WT and Esel KO mice were treated with 50μg lipopolysaccharide (LPS) from *E Coli* 0111:B4 (Sigma-Aldrich), made up to 200μL volume in normal saline, by *intraperitoneal* (*ip*) injection to induce systemic inflammation [5].

### Immunohistochemistry

Immunohistochemistry was performed on acetone-fixed cryosections of freshly harvested hearts of the WT (with/without LPS pre-treatment) and Esel KO (LPS pre-treated) mice, using a standard protocol detailed in the S1 File. The primary antibodies used were MES-1 (for Esel), MEC13.3 (for PECAM-1, an endothelial marker) and rat IgG2a,κ isotype negative control mAb. Color was developed using 3,3′-diaminobenzidine with hematoxylin counterstaining.

### Reverse Transcriptase—Real Time Quantitative Polymerase Chain Reaction (RT-qPCR)

WT mice were pre-treated with LPS as described above. The duration between LPS treatment and animal sacrifice for immediate tissue harvesting was noted as the LPS_Time_. RNA extraction and RT-qPCR for Esel and hypoxanthine phosphoribosyltransferase-I (HPRT-I, a house keeping gene) in the hearts were performed; only apical-half of the harvested hearts were used in order to avoid measuring Esel from the great vessels. Note that Esel is expressed on endothelial cells and not on other cell types. RT-qPCR was performed using standard protocols and kits according to the manufacturers’ instructions. All PCR reactions were carried out in triplicates on a 96-well plate. Means of the replicates were used. Esel mRNA concentration was expressed as % HPRT-I. Further details in S1 File.

### Microbubble Preparation

A generic (non-conjugated) microbubble was first produced by sonicating C_3_F_8_-sparged aqueous suspension containing 1,2-distearoyl-*sn*-glycero-3-phosphocholine (DSPC; Avanti Polar Lipids, AL), 1,2-distearoyl-*sn*-glycero-3-phosphoethanolamine-N-(maleimide(polyethylene glycol)-2000) (DSPE-PEG2000-Maleimide; Avanti Polar Lipids), mono-stearate poly(ethylene)glycol (PEG40-stearate; Sigma-Aldrich), and fluorescent dye 1,1'-dioctadecyl-3,3,3',3'-tetramethylindocarbocyanine perchlorate (DiI; Molecular Probes) at 75:9:14:2 molar ratio. Reduced MES-1 F(ab’)_2_ containing 2 thiol groups per F(ab’)_2_ (prepared as described in S1 File) were then linked to maleimides on the bubble shell outer-surface by maleimide-thiol conjugation to produce the Esel targeting bubble. The conjugation reaction ratio was 4.338x10^6^ F(ab’)_2_ molecules per bubble, estimated F(ab’)_2_:maleimide reaction molar ratio ≥10:1 (see S1 File). The reaction was terminated by adding excess N-Ethylmaleimide (NEM, Sigma-Aldrich) to quench any unreacted thiols. Bubbles before and after conjugation were washed with cold degassed normal saline several times, by centrifugation-flotation with exchange of the subnatents, to remove unincorporated components (including excess F(ab’)_2_ and NEM) and bubble fragments. In previous experiments, we found that by keeping the conjugation sites on each ligand to a minimum (ie, reducing only 1 inter-chain disulfide bond to produce 2 thiols per F(ab’)_2_), and inactivating any unreacted thiols after bubble conjugation (eg, alkylation of unreacted thiols using NEM), significant bubble aggregation due to cross-linking was avoided [2]. Further details in S1 File.

### Microbubble Microscopy, Concentration, Size Distribution and Charge Analysis

Bubbles were diluted (eg, 1:200) in cold normal saline and examined under microscopy in a hemocytometer. Bubble concentration and size distribution were determined by electrozone sensing in a Coulter Multisizer IIe equipped with a 30μm-diameter orifice counting tube (Coulter Electronics), according to the manufacturer’s instructions. The electrozone sensing has detection range 0.72–18μm, resolution 0.09μm. All bubble concentrations and calculated dosages in this study were based on electrozone sensing (nb, different methods such as microscopy, electrozone sensing or laser diffraction, give different particle concentration and size distribution [6], due to differences in the nature of the analytical process and the minimum size detectable). For bubble charge analysis, the bubbles were dispersed in 1mL of 1mmol/L KCl (pH 7.4) at ≈10^7^ bubbles/mL as described by others [7, 8]. Bubble net charge was determined as the zeta potential by light scattering in a Zetasizer Nano ZS (Malvern Instruments), according to the manufacturer’s instructions.

### Targeting Microbubble Binding Assay *In Vitro*

100μL of Esel targeting or non-targeting (generic) bubbles at 2.5x10^7^ bubbles/mL were placed on inverted polystyrene petri-dishes coated with 200μL of recombinant homodimeric mouse Esel protein (R&D Systems) at 7nmol/L (dish E), or on Esel coated dishes previously blocked with 500μL of excess MES-1 F(ab’)_2_ at 67nmol/L (dish B), or on non-coated dishes where phosphate buffered saline pH 7.5 (PBS) was used instead of Esel for dish coating (dish P). Unattached bubbles were gently washed off after 1min. The dishes were then re-filled with cold degassed PBS for immediate examination under an upright light microscope equipped with immersion objective lens. The number of bubbles attached on each dish was counted and averaged from 10 random optical fields (OFs) to determine the attached bubble density. Further details in S1 File. Different batches of bubbles (made at different times) were tested on different occasions. To correct for experimental variations amongst the different occasions, the attached bubble density was normalised against that of the targeting bubbles on dish E in the corresponding occasion.

### Intravital Microscopy of Esel Targeting Microbubbles in the Mouse Cremaster

WT and Esel KO mice were pre-treated with 50ng recombinant IL-1β (R&D Systems) intrascrotally to induce cremaster inflammation, 2 hours (h) before general anesthesia (xylazine/ketamine mixture *ip*) and exteriorization of the muscle for intravital microscopy. 1.5x10^7^ Esel targeting bubbles in 100μL normal saline were administered as a rapid *iv* bolus through the tail vein catheter of these animals, followed by a 100μL normal saline flush, for intravital microscopy in the cremaster. Observations were made using an upright microscope equipped for bright-field and fluorescence microscopy, with 20x and 40x immersion objective lens, charge-coupled device (CCD) and silicon intensifier target (SIT) cameras. See S1 File for detailed set-up. Blood flow and bubbles were assessed over several OFs encompassing a number of different vessels in different vascular beds (arteries, veins, capillaries) under bright-field and fluorescence microscopy. The number of freely circulating bubbles in a monitor OF were counted over 10s under fluorescence microscopy at 5, 7, 10 ± 15min after bubble injection. The accumulation of attached bubbles (defined as not moving for >3s) in an OF field were assessed for up to 15min post bubble injection. At ≈15min (when freely circulating bubbles were absent/minimal), multiple OFs were used to assess the number of attached bubbles in 20–40μm diameter venules: one to five 400μm-length segments of 2–6 venules were examined per animal. In some animals, the attached bubbles in the same OF were assessed for up to 90min under intermittent combined bright-field and fluorescence microscopy, looking for cellular internalization or transmigration into the tissue interstitium. Shear rates against bubble attachment were determined from microvascular center-line red blood cell velocities (*V*_rbc_) in 5 random segments of 20–40μm diameter venules, using an Optical Doppler Velocimeter (Microcirculation Research Institute, Texas A&M University, Texas), before bubble injection. All animals were sacrificed at the end of the experiment. For analysis, the density of attached bubbles was expressed as the number of bubbles per vessel surface area (VSA), where VSA = π*DL*, *D* and *L* are the vessel segment diameter and length, respectively. The attached bubble density for each venule was taken as the mean of its segments, and that for each animal was taken as the mean of its venules. Shear rate was calculated using: $\text{Shear}\;\text{rate}=8\frac{V_{\text{b}}}{D}$, where $V_{\text{b}}=\frac{V_{\text{rbc}}}{\alpha }$, *V*_b_ is the mean bulk velocity, *α* is the factor converting *V*_rbc_ to *V*_b_ (taken as 1.6 for Poiseuille flow in veins). The shear rate for each animal was taken as the mean of the 5 random venule-segments sampled.

### Confocal Microscopy of Esel Targeting Microbubbles in the Mouse Cremaster

1.5x10^7^ Esel targeting bubbles were administered to WT and Esel KO mice pre-treated 2.5h before with 50ng IL-1β intrascrotally, as described in intravital microscopy. 15min post bubble injection, a rapid *iv* bolus of a 150μL cocktail containing 50μg AF488-MES-1 (against Esel) + 25μg allophycocyanin-labelled mAb (against PECAM-1, an endothelial marker) in normal saline was administered, followed by a 100μL normal saline flush. After a further 15–20min, animals were given terminal anesthesia by xylazine/ketamine mixture *iv*, followed by manual perfusion with PBS to remove unattached bubbles and mAb from the circulation. Immediately thereafter, the cremasters were harvested and fixed in 4% paraformaldehyde PBS for confocal microscopy. 3 different fluorescence were scanned: DiI for bubbles, Alexa Fluor 488 for Esel and allophycocyanin for PECAM-1. Further details in S1 File.

### Ultrasound Imaging

15 WT and 8 Esel KO mice were imaged. All were pre-treated with LPS. Ultrasound imaging was performed under *ip* xylazine/ketamine general anesthesia. The Acuson Sequoia 512 clinical ultrasound scanner equipped with a 15L8-s linear array transducer (Siemens, CA) was used. Gel was coupled between the shaven skin and the transducer. 14MHz contrast pulse sequencing (CPS) mode imaging at low power (mechanical index (MI) = 0.22–0.26), dynamic range 55dB was used. Gain and other settings were fixed. Bubble signals were presented in heated object scale (‘CPS-contrast only’ images), tissue signals in grey scale (‘B-mode’ images). Baseline images of the heart in the parasternal short axis (PSA) papillary muscle level, parasternal long axis (PLA) and apical 4-chamber (A4C) views were acquired before bubble administration. Imaging was then maintained in the PSA view by fixing the transducer in position with a free standing clamp. A stopwatch was started and 10^8^ Esel targeting bubbles (in 100μL volume made up with normal saline) injected at 10s as an *iv* bolus over 1–2s through a cannula in the tail vein. This was followed by a 100μL normal saline flush at 20s. *To capture real-time sequence of events and detect Esel expression in the heart*, continuous ultrasound imaging was performed and recorded as 3s-digital clips, starting at time 0 on the stopwatch and repeated at pre-determined time intervals. *To determine the nature of bubble signal attenuation*, the following was performed in addition: (i) other views of the heart (PLA, A4C) were acquired at the end; (ii) 7MHz CPS imaging at MI 0.22 (keeping the gain & other settings the same as 14MHz imaging) was acquired at baseline & end of the 14MHz imaging in some animals; and (iii) wider PSA view to include other structures surrounding the heart was acquired at 5min intervals. *To image extra-cardiac tissues*, 14 and 7MHz CPS imaging of the thorax, abdomen and pelvis were also performed in the antero-posterior plane at baseline & end of the cardiac imaging study in some animals. To do this, the probe was positioned transversely and moved slowly caudal from just below the neck to the pelvis during image recording. All animals received only one dose of bubbles to negate carry-over effects from previous bubble dosing (eg, blocking of Esel binding sites). The duration between LPS treatment and the administration of bubbles was noted as the LPS_Time_. All animals were sacrificed at the end. Further details in S1 File.

### Acoustic Quantification of Esel Expression in the Heart

As circulating bubble signal in the left ventricular (LV) cavity (blood pool) was absent/minimal by ≈20min post bubble administration in all animals, the bubble signal intensity in the myocardium at 24min was assessed for acoustic quantification of Esel expression in the heart. To do this, several end-diastolic image frames of the heart (‘CPS-contrast only’ images) within the 3s-recording period at 24min 10s post bubble administration were selected and aligned. A videodensitometric method was used to quantify the CPS bubble signal intensities in the anterior wall of the myocardium (region of interest M), using the YABKO software (Charlottesville, Virginia). This region was chosen because it was consistently least/minimally affected by ultrasound attenuation in all animals. The CPS video signal intensities (*VI*’s) were ‘linearized’ by log-decompression using the formula: $\text{Linearized}\;VI\;=255\times 10^{\left(\frac{VI-255}{255}\times \frac{\text{Dynamic}\;\text{Range}}{20}\right)}$. The linearized *VI* (*I*) was expressed in arbitrary acoustic units (AU). *I*’s of the images were averaged, then subtracted by average *I* of the baseline (before bubble administration) images. The baseline-subtracted *I* in the myocardium at 24min 10s post bubble administration (*R24*) represented the retained bubble signal intensity in the myocardium at 24min 10s post bubble administration. As the circulating bubble signal intensities in the myocardium at 24min 10s were negligible (ie, a circulating bubble signal intensity of <0.1 AU in the LV cavity (blood pool) at this time would contribute an undetectable circulating bubble signal intensity of <0.005–0.024 AU in the myocardium, due to a relative myocardial blood volume of 5–24%) [9–11], their subtraction from *R24* was not required. For data analysis, *R24* was correlated against the level of Esel expression in the heart, in terms of LPS_Time_ or Esel mRNA concentration by qRT-PCR. Note: (i) The Esel mRNA concentration was determined from a standard curve of LPS_Time_ *vs* Esel mRNA concentration in the hearts of 42 mice (LPS_Time_ range: 3–16h) [2]. Due to the relatively long duration of each imaging study (mean ≈45–60min from the time of bubble administration to tissue harvesting), it was not possible to correlate *R24* of individual animals against its own Esel expression level determined *ex-vivo* by independent means (eg, RT-qPCR), because Esel expression decreased rapidly with time in the mouse model used [2, 5], such that the retained bubble signal intensities reflected Esel expressions around the time of bubble administration rather than tissue harvest. (ii) We have shown previously that the relationships between the cell-surface Esel protein (actual bubble target) concentration and LPS_Time_ (for LPS_Time_ ≥3h) or Esel mRNA concentration in the heart were curvilinear; and approximately linear in the range of LPS_Time_’s (4–6h) or mRNA concentrations (50–220% HPRT-I) imaged in this study [2, 5]. (iii) As Esel expression in the myocardium was essentially global and uniform in the mouse model used [2], the quantification of Esel expression from the anterior myocardial wall by ultrasound, and from the apical-half of the heart by qRT-PCR, were regarded as equivalent (both represented that of the whole heart).

### Statistics

Pearson correlation was performed, where indicated. Student’s t-test or one-way ANOVA with Tukey’s post-hoc analysis was used for significance testing, where appropriate, with *p*<0.05 taken as statistically significant.

## Results

### Esel Targeting Microbubbles

**Fig 1:**
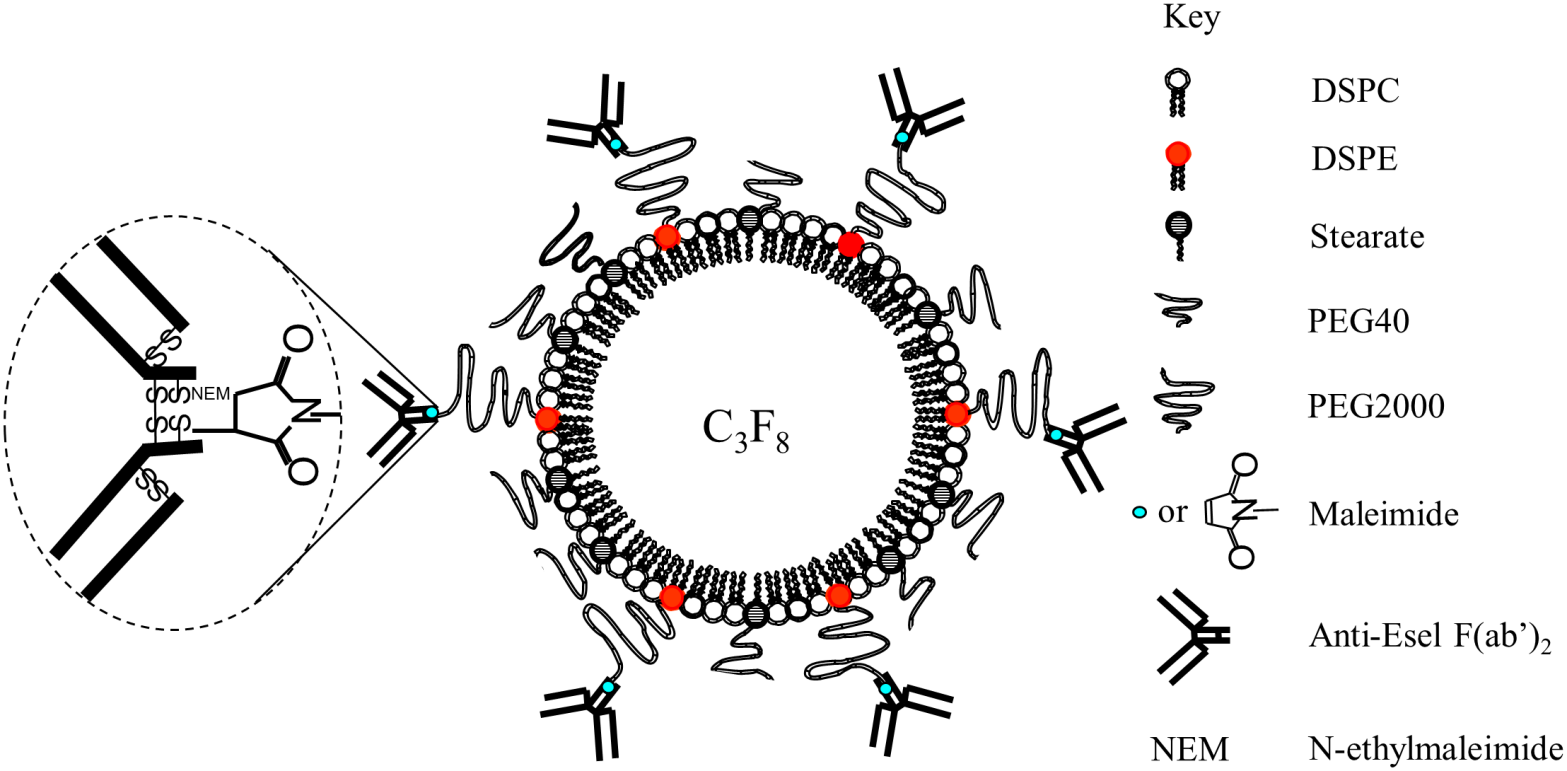
Schema of Esel targeting microbubble.

**Fig 2:**
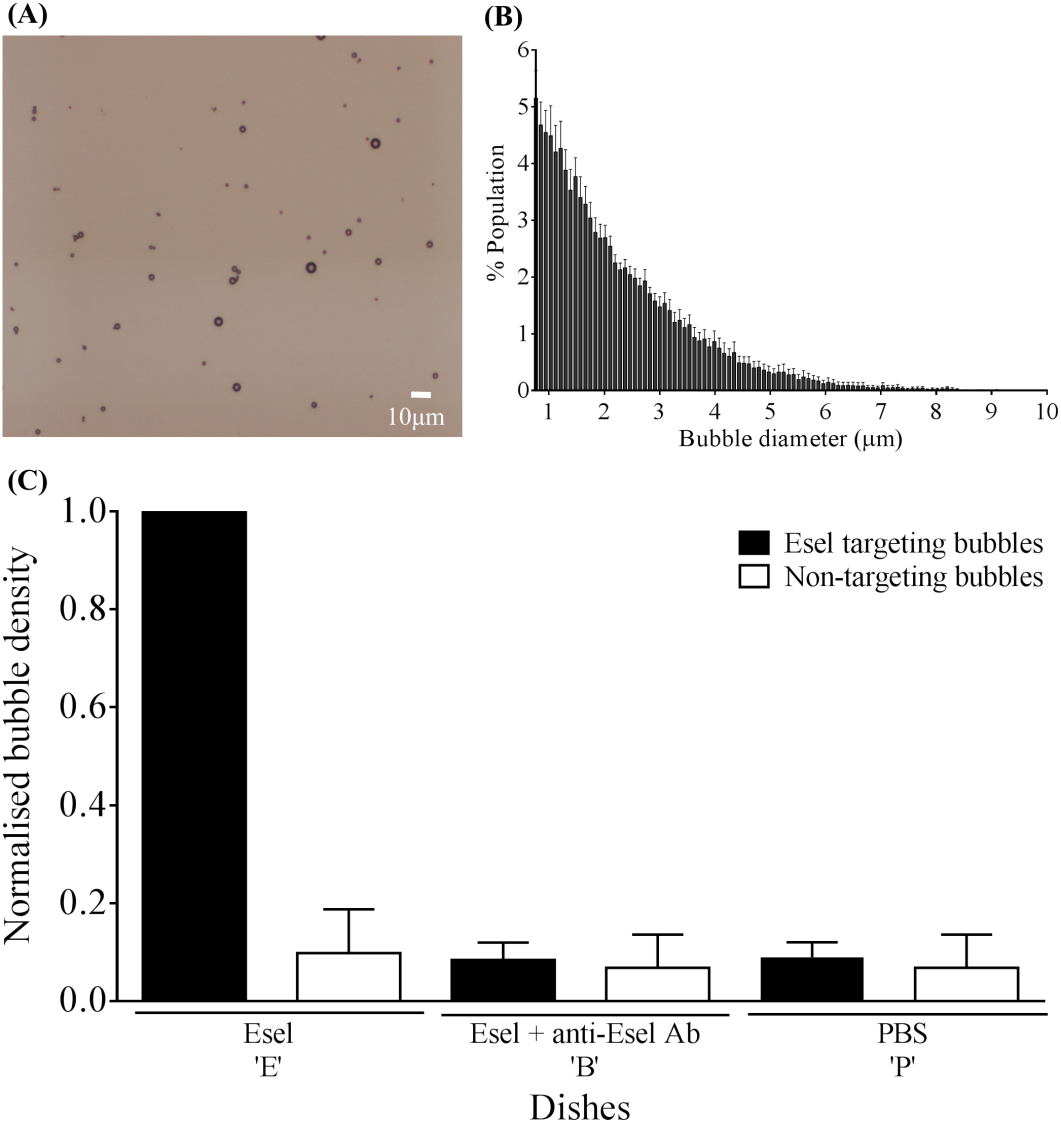
*In vitro* validation of Esel targeting microbubbles. (A) Bright-field microscopy of Esel targeting bubbles. (B) Size distribution of Esel targeting bubbles. Plotted are the mean and SEM (error bar) for 5 batches of bubbles prepared on separate occasions. (C) *In vitro* binding of Esel targeting bubbles. Plotted are the mean and SEM (error bar) of attached bubble densities relative to that of Esel targeting bubbles on dish E, n = 5 batches of Esel targeting bubbles and 2 batches of non-targeting bubbles.

A schema of the Esel targeting bubbles is shown in Fig 1. The bubbles showed spherical morphology; bubble-bubble aggregation or cross-linking was minimal as assessed qualitatively on microscopy, Fig 2A. The bubble size distribution was reproducible amongst 5 batches prepared on separate occasions; the mean (SEM) bubble diameter was 2.2 (0.2) μm, 98.6% or 100% of the bubble population were under 6 or 10μm in diameter, respectively, Fig 2B. The bubbles had a near neutral charge, zeta potential = 5mV at pH 7.4.

### *In Vitro* Binding of Esel Targeting Microbubbles

Esel targeting bubbles attached to Esel coated on dish (dish E): the mean (SEM) attached bubble density was 2060 (1070) bubbles/mm^2^ for the 5 batches of targeting bubbles produced on separate occasions, Fig 2C. The specificity of the targeting bubbles to Esel was demonstrated by the abolishment of bubble attachment when Esel was pre-blocked with excess anti-Esel antibody (F(ab’)_2_) (dish B). Additional negative controls using targeting or non-targeting bubbles (generic non-conjugated bubbles) on dishes not coated with Esel (dish P), or non-targeting bubbles on dish E and B showed similar low levels of bubble attachment. One-way ANOVA with Tukey’s post-hoc analysis showed significant differences between targeting bubbles on dish E and the negative controls (*p*<0.0001); no significant difference was observed amongst the negative controls.

### *In Vivo* Validation of Esel Targeting Microbubbles

**Fig 3:**
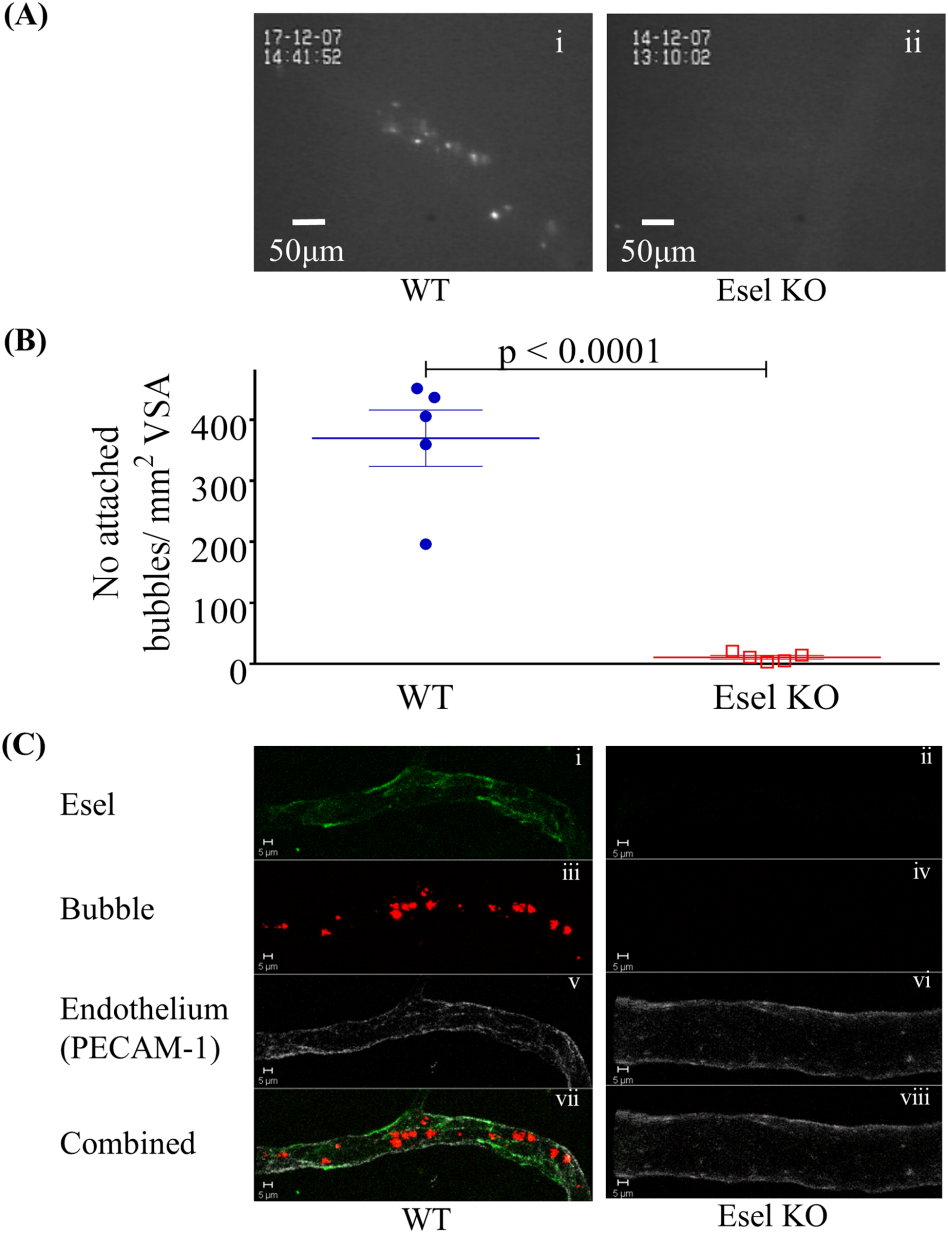
*In vivo* validation of Esel targeting microbubbles. Intravital microscopy of Esel targeting bubbles in the mouse cremaster. (A) Fluorescence microscopy (SIT camera images, magnification 200x) of attached bubbles in a representative WT (i) and Esel KO (ii) mouse of Fig 3B (S1 and S2 Videos). (B) Quantification of bubbles attached to the cremaster venule. Each point represents one animal; group mean and SEM (error bars) are shown; n = 5 WT and 5 Esel KO. (C) Confocal microscopy of Esel targeting bubbles in the mouse cremaster venule. Esel in green (i, ii), bubbles in red (iii, iv), endothelium (PECAM-1) in grey (v, vi), all 3 components combined (vii, viii).

Esel targeting bubbles were administered to 5 WT (body weight mean (SD, range): 25 (2, 23–27) g) and 5 Esel KO (24 (2, 22–27) g) mice at 3.1–4.1h and 3.3–4.5h post IL-1β treatment, respectively. Under intravital microscopy, the bubbles were seen to circulate and reach the cremaster muscle ≈7–17s post bubble administration. The bubbles attached and accumulated in the cremaster venules of the WT mice (mean (SEM) attached bubble density = 370 (46) bubbles/mm^2^ VSA), this was minimal in the KOs (11 (3) bubbles/mm^2^ VSA), *p*<0.0001 (student’s t-test), suggesting high targeting specificity and low degree of non-specific bubble attachment/retention, Fig 3A and 3B (S1 and S2 Videos). Bubble attachment in the arteries, arterioles, capillaries or large veins was absent/minimal. Rolling was observed in a small minority of bubbles; complete detachment of the attached bubbles was infrequently seen. Intravascular obstruction by the bubbles and bubble attachment to leukocytes were not detected. Transmigration into the tissue interstitium or cellular internalization of the attached bubbles was also not detected, when observed for up to 90min.

Confocal microscopy showed co-localization of the targeted bubbles with endothelial cell-surface Esel in the WT cremaster venules (n = 3 animals, body weight 21.8–24g), further confirming Esel specificity of the targeting bubbles, Fig 3C. Esel expression was not detected in the KOs (n = 2, body weight 25.7–28.4g).

The shear rates against bubble attachment were not significantly different between the WT (mean (SEM) = 329 (46) s^-1^, n = 3) and KO (211 (37) s^-1^, n = 3) mice, *p* = 0.30 (student’s t-test). The diameter of the vessels sampled for shear rates were similar between the two groups: mean (SEM) vessel diameter 30 (3) μm in the WT *vs* 34 (1) μm in KO group, *p* = 0.12 (student’s t-test). The mean body weight (SD, range) was 26 (2, 25–28) g and 27 (1, 26–29) g in the WT and KO group, respectively.

**Fig 4:**
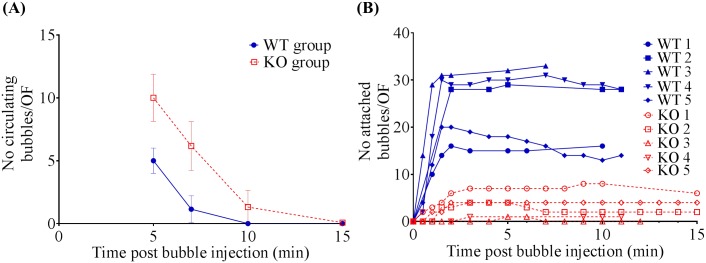
*In vivo* kinetics of Esel targeting microbubbles. Intravital microscopy of Esel targeting bubbles in the mouse cremaster. (A) Elimination kinetics of circulating bubbles. Group mean and SD (error bars) are plotted. (B) Accumulation kinetics of attached bubbles. N = 5 WT and 5 KO in both (A) & (B); these are the same animals as those in Fig 3A and 3B.

Fig 4A showed that the number of freely circulating bubbles decreased exponentially with time (the exponential nature supported first-order kinetics [12] in the elimination of the bubbles). Circulating bubbles cleared from the blood pool sooner in the WTs than KOs, presumably due to there being less circulating bubbles in the WTs, because a larger proportion of the administered bubbles ended up as non-circulating bubbles due to their attachment to Esel in the WTs. Circulating bubbles that do not become attached are eliminated by the reticuloendothelial tissues (eg, liver, spleen) and lungs. In all animals, the circulating bubbles were minimal/undetectable beyond 10min post bubble administration. The density of the attached bubbles on the cremaster venules increased with time, reaching a plateau soon after (≈2min) *iv* bolus administration of the bubbles, Fig 4B. In one WT animal (WT 3), excessive and prolonged light exposure in the OF was inadvertently applied >7 min post bubble administration—this reduced subsequent fluorescence detection of the bubbles retained in that OF by the SIT camera. As a result, bubble counts >7 min post bubble administration in that OF were unreliable and therefore excluded from analysis in this animal.

### Ultrasound Imaging

**Fig 5:**
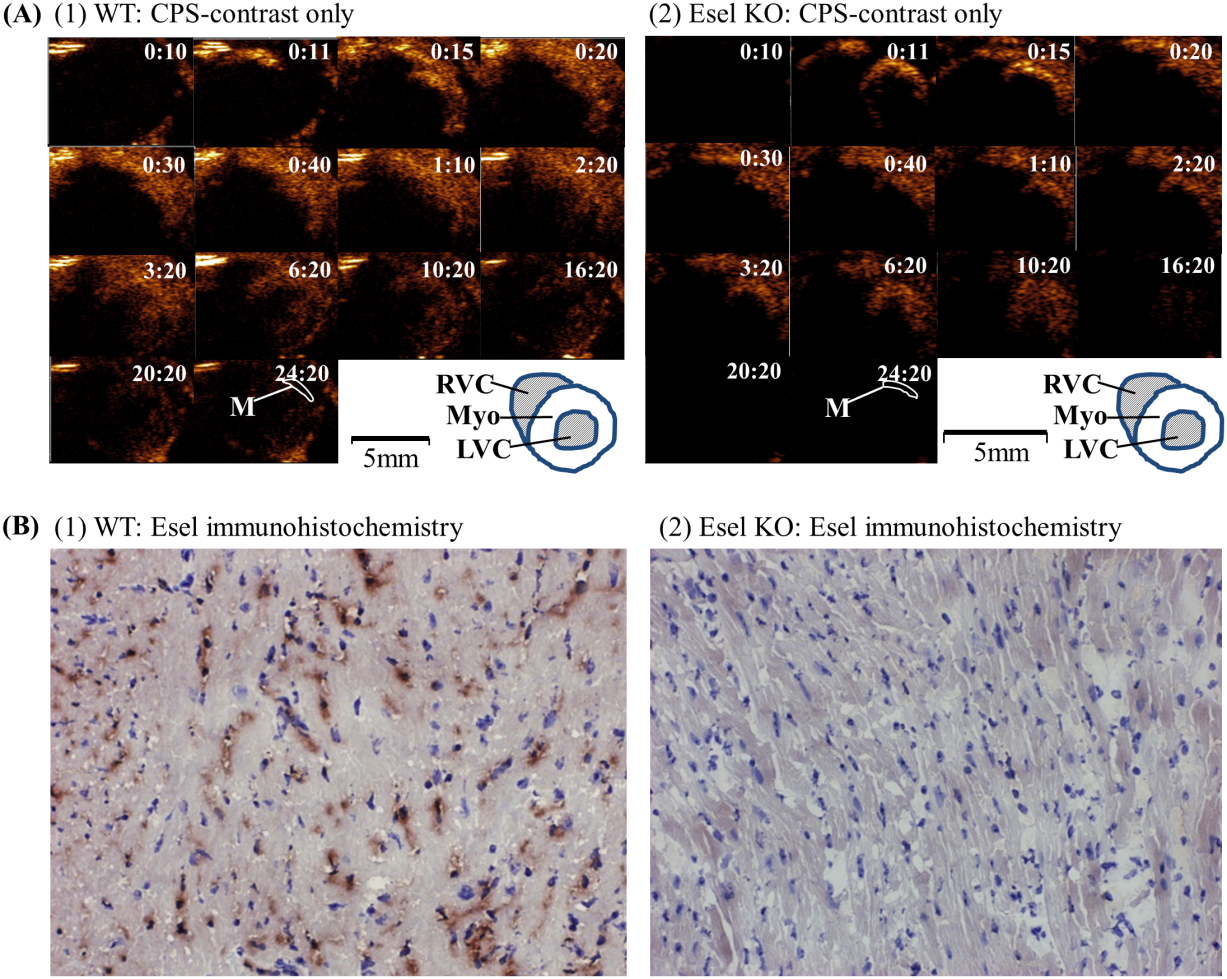
Real-Time Ultrasound Molecular Imaging of Esel Expression in the Mouse Heart. (A) Sequential 14MHz CPS images of the heart in end-diastole PSA view from 00:10 to 24:20 post *iv* bolus administration of Esel targeting bubbles, in a (1) WT and (2) Esel KO mouse pre-treated with LPS, respectively. Bubble signal is presented in a heated object scale. M: region of interest in the myocardium for acoustic quantification. A labelled diagram of the ultrasound images is shown: LVC (left ventricular cavity), Myo (left ventricular myocardium), RVC (right ventricular cavity). (B) Frozen section immunohistochemistry for Esel in the heart of a (1) WT and (2) Esel KO mouse. Positive staining = brown color. Controls for staining is shown in Fig A in S1 File. Magnification 200x.

**Fig 6:**
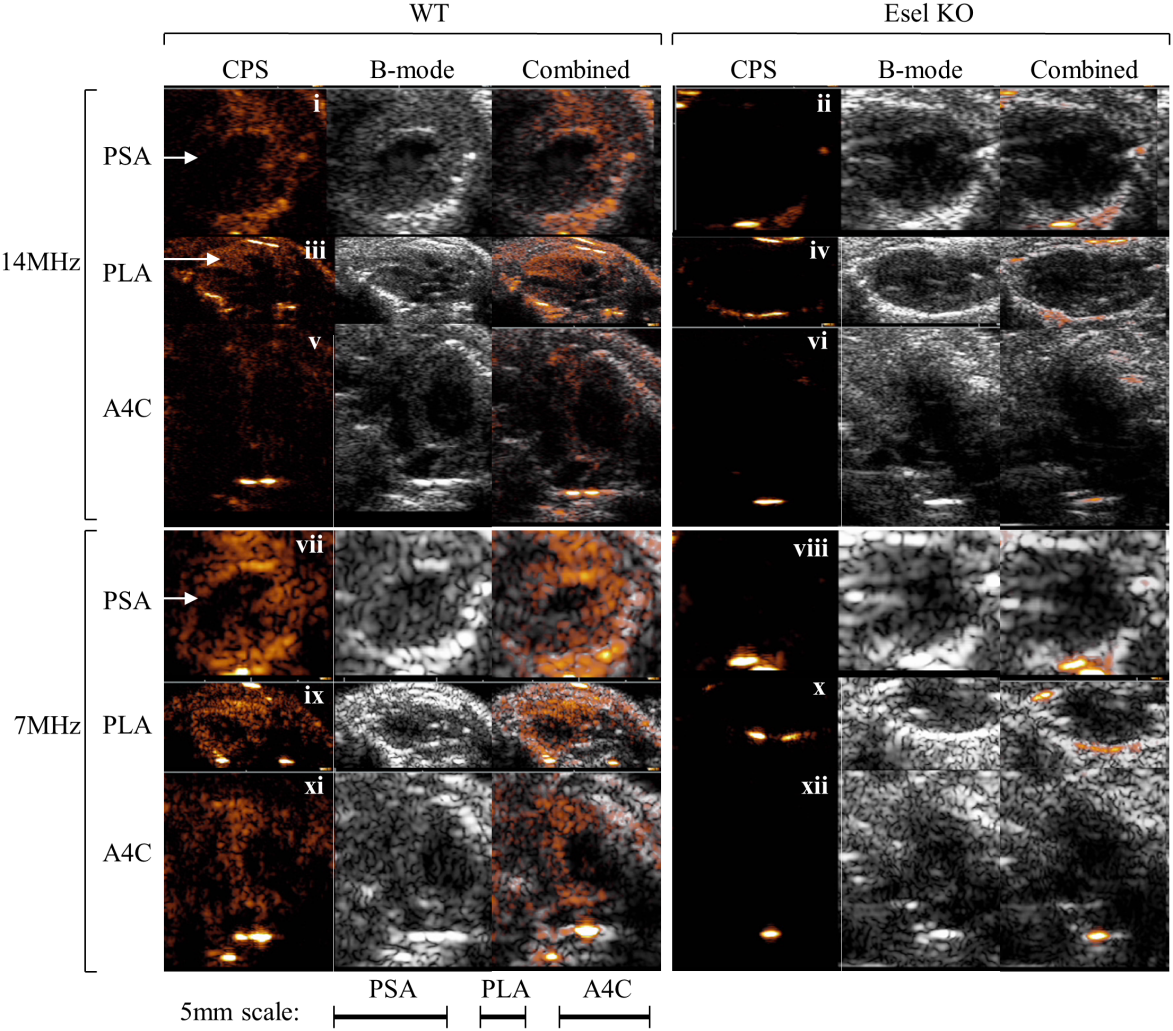
Real-Time Ultrasound Molecular Imaging of Esel Expression in the Mouse Heart. PSA, PLA and A4C views of the heart at 14 and 7MHz CPS, >20min post bubble administration (when freely circulating bubbles have cleared from the blood pool (LV cavity)). Animal, gain and MI were the same between both frequencies. Bubble signal is presented in a heated object scale. Arrow indicates recovery of retained bubble signal in the mid anteroseptal wall by changing the scan plane from PSA to PLA or ultrasound frequency from 14 to 7MHz. Baseline images before bubble administration are shown in Fig B in S1 File.

**Fig 7:**
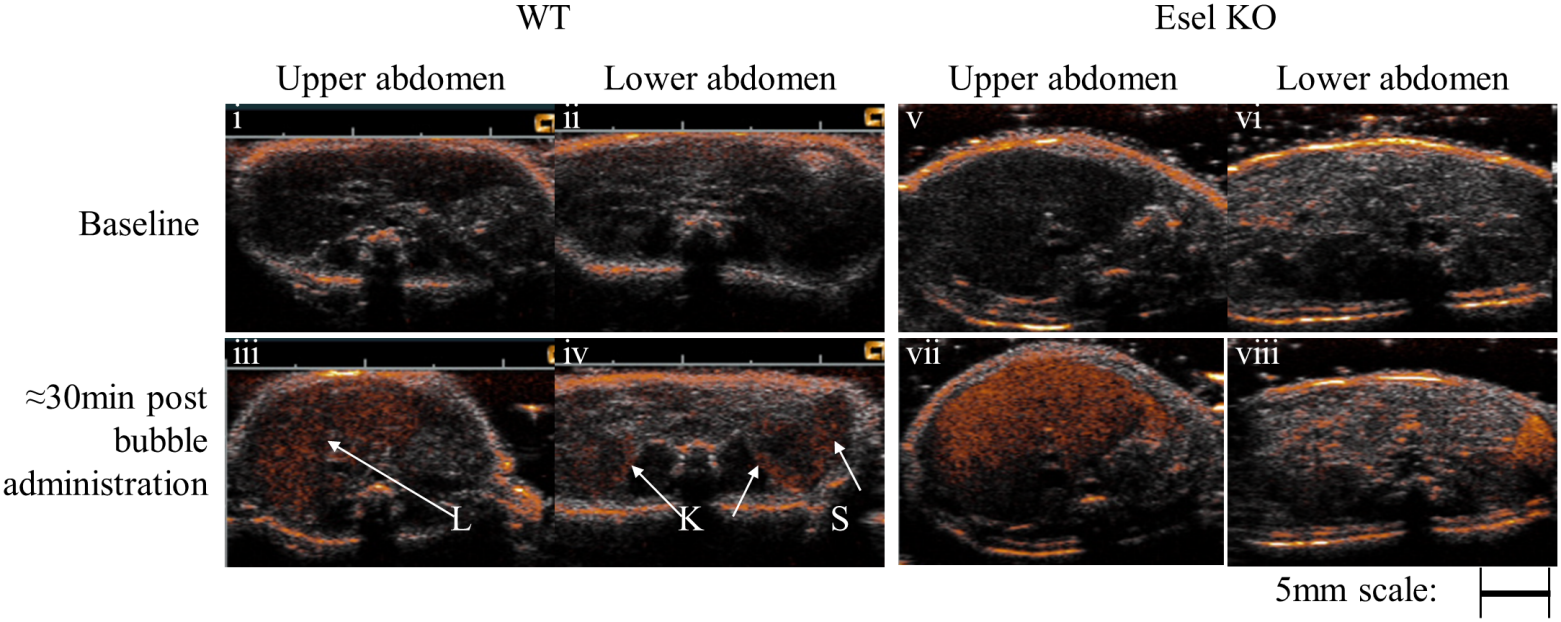
Real-Time Ultrasound Molecular Imaging of Esel Expression in the Mouse Abdomen. Combined 14MHz ‘CPS-contrast only’ and ‘B-mode images’ before (i, ii, v, vi) and ≈30min post (iii, iv, vii, viii) bubble administration. ‘CPS-contrast only’ = bubble signal intensity in heated object scale. ‘B-mode’ = tissue signal intensity in grey scale. Arrows indicate the kidneys (K), liver (L), or spleen (S).

**Fig 8:**
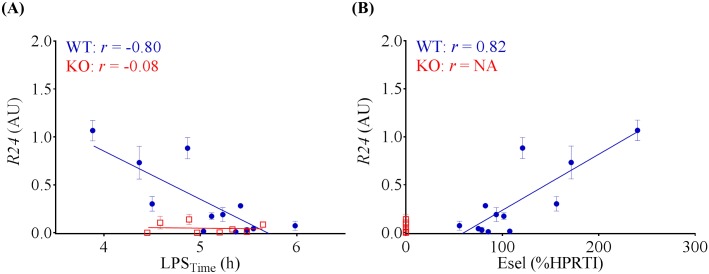
Acoustic Quantification of Esel Expression. Acoustic signal intensity of targeted bubbles retained in the WT myocardium (*R24*) correlated with the level of Esel expression. WT (blue); KO (red); not applicable (NA). *R24* is plotted as mean with SD (error bars). Pearson *r* (*r*) is shown. WT mean (SEM, range) = 0.32 (0.11, 0.01–1.07) AU, n = 12. KO mean (SEM, range) = 0.05 (0.02, 0–0.14) AU, n = 8.

1. **Animals**. 15 WT and 8 Esel KO mice were imaged. 3 WT mice were excluded from analysis because of bubble dosing error (one animal) or uncertainties regarding the level of Esel expression (two animals). Thus, 12 WT (mean body weight (SD, range) = 19.7 (1.4, 18–22) g) and 8 KO (22.3 (3.7, 17–28) g) mice were analysed. The LPS_Time_ ranged 3.9–6.0h in the WT and 4.5–5.7h in the KO group.
2. **Cardiac Imaging**. Bright signal artefacts present both before and after bubble administration could be seen in the WT and Esel KO animals; these artefacts were small and outside the myocardium, Figs 5A and 6, and Fig A in S1 File.
  The real-time sequence of events in the heart for a single *iv* bolus of the targeting bubbles is shown in Fig 5a. Bubble signal was detected first in the right heart chambers within 4 heart beats (≈1s) of bubble administration. This was followed by bubble signal appearing in the left heart chambers as the bubbles returned from the pulmonary circulation. The bubble signal intensities rose rapidly, peaking in the LV cavity and myocardium within 6–7 heart beats (≈1.5–2s) and 9–12 heart beats (≈2–3s) after bubble administration, respectively. The signal intensities then decreased over time.
  Esel expression in the WT myocardium was visualized in real-time, best demonstrated by the persistence of bubble signals (retention of attached bubbles) in the myocardium beyond the clearance of circulating bubbles from the blood pool (LV cavity). In the KO myocardium, the persistence of bubble signals was minimal, consistent with a minimal degree of non-specific bubble retention, Figs 5A and 6.
  Frozen section immunohistochemistry confirmed the presence and absence of Esel expression in the LPS pre-treated WT and KO hearts, respectively. The spatial distribution of Esel expression was essentially uniform throughout the myocardium, but limited to the capillaries and post-capillary venules, Fig 5B and Fig A in S1 File.
  Significant ultrasound attenuations due to the high initial bubble concentrations occurred early following bubble bolus administration, with major loss of signals in regions of the heart located distally in the ultrasound path (eg, 5–10 o’clock positions in the myocardium and adjacent LV cavity in the PSA view, Fig 5A). As the circulating bubble concentrations in the blood pool decreased over time, the attenuations diminished (compare frame 0:30 *vs* 10:20 in Fig 5A) but did not disappear (frame 20:20 in Fig 5A1)—most likely due to attenuations caused by overlying bone, lung air ± retained bubbles. This caused pseudo-loss of targeted bubble signals for Esel in the WT mice. However, by changing the scan plane to alter the relative positions of the overlying entities, or by lowering the ultrasound frequency to increase its penetrative depth, these attenuations could be overcome with good ‘recovery’ of the retained bubble signals, Fig 6. The global expression of Esel in the WT myocardium was thus demonstrated on ultrasound imaging, consistent with the immunohistochemistry findings.
3. **Imaging of Other Tissues**. Comparison of the thoracic, abdominal and pelvic scans between the WT and Esel KO animals with reference to the baseline images (before bubble administration), showed low/minimal non-specific retention of the targeting bubbles in more than one non-reticuloendothelial tissues. Esel expression was detected in the renal cortex of the WT but not KO animals (Fig 7)–the spatial distribution of the targeted signals was consistent with the predominant expression of Esel in the glomeruli [13]. As expected, the targeting bubbles were taken up by the spleen and liver in both WT and KO animals, the major reticuloendothelial tissues involved in bubble elimination.
4. **Acoustic quantification of Esel Expression in the Heart**. Acoustic quantification of the level of Esel expression to a high degree of quantification was possible using *R24*, which correlated strongly with the level of Esel expression in terms of LPS_Time_ (r = -0.8) and Esel mRNA concentration (r = 0.82) in the heart, Fig 8. The latter two previously shown to be linearly related to the cell-surface Esel protein (actual bubble target) concentration in the heart, in the expression range imaged [2].

#### Adverse Effects

No acute adverse effects were observed in all 38 animals following bubble administration: intravital microscopy (5 WT, 5 KO), confocal microscopy (3 WT, 2 KO) and ultrasound molecular imaging (15 WT, 8 KO).

## Discussion

In this early developmental study, a targeting bubble based on maleimide-thiol conjugation chemistry with the generic (non-targeting components) composition was successfully engineered. The bubble demonstrated all three of the following properties desired for clinical applications: (i) low degree of non-specific retention in more than one non-reticuloendothelial tissue; (ii) effective for ultrasound imaging in real-time; and (iii) effective for acoustic quantification of the targeted molecule to a high degree of quantification. Based on the maleimide-thiol conjugation chemistry and properties (i)-(iii), the bubble is a potential candidate for further development towards clinical translation.

The generic composition of our bubble consisted of C_3_F_8_-gas encapsulated within a phospholipid-shell made from an aqueous suspension of DSPC:DSPE-PEG2000-Maleimide:PEG40-stearate at 75:9:14 molar ratio (a small amount of DiI fluorescent dye was added for bubble visualization under intravital/confocal microscopy). The same composition has not been reported in other bubbles, although frequently such information is not available or insufficiently disclosed. Where known, the composition differs to ours in the number, type, combination, or molar ratio of the chemical components used (Table A in S1 File).

The maleimide-thiol conjugation chemistry has the potential advantage of low immunogenicity. Anti-maleimide linker immune response has been detected where maleimide derivatives containing certain chemical groups associated with the maleimide function were used, eg, 4-(*p*-maleimidophenyl)butyrate (MPB), *m*-maleimidobenzoate (MBS), or 4-(*N*-maleimidomethyl)-cyclohexane-l-carboxylate (MCC) [14–16]. In this study, we used (simple) maleimides (present in DSPE-PEG2000-Maleimide and NEM) in preference to maleimide derivatives, to avoid immunogenic/toxic side-effects due to the presence of additional/unnecessary chemical groups associated with the maleimide linker. In clinical translation, conjugate products containing maleimide or even maleimide derivative (o-phenylenedimaleimide) such as the Hemospan (a conjugated haemoglobin (MP4) blood substitute) [17] or the conjugated saporin (used in cancer treatment) [18], respectively, have been tested in humans without significant immunogenic/toxic side-effects attributable to the maleimide linker *per se*. Hemospan has completed Phase 3 clinical trial [17]. Nonetheless, formal immunogenicity and toxicity studies of our bubble will be required. The thioether bond formed between maleimide and thiol is strong and rapid at near neutral pH (bond strength in the order of nanonewtons; second-order rate constant 0.8–1.2x10^4^ M^-1^s^-1^) [19]. The near neutral pH is advantageous in avoiding negative impact on the ligands and bubbles during the conjugation process, and preventing dissociation of the ligands from the bubbles *in vivo*.

The lipid component containing the conjugation functional group generally makes up 1–5 mol % of the bubble composition, range 1–20 mol % (Table A in S1 File). Our bubble falls into the mid high range with 9 mol%, giving an estimated ≈30k/μm^2^ bubble surface area or ≈400k/bubble of maleimide functional groups available for linking with targeting ligands (calculation on page 7 of S1 File). The average of quoted targeting ligand densities in the literature is 5–30k/μm^2^ bubble surface area (range 0.6–86 k/μm^2^) or 100–400k/bubble (range 3–820 k/bubble), Table A in S1 File. Thus, our bubble contained enough maleimide groups for conjugating a sufficient number of targeting ligands onto the bubble surface for efficient target binding under flow conditions, as demonstrated in this study. However, it should be noted that amongst various factors, a bubble’s target binding efficiency is also dependent on the targeting ligand’s binding kinetics and valency.

The conjugation reaction ratio used to produce the successful Esel targeting bubble in this study was 4x10^6^ F(ab’)_2_ molecules per bubble, the estimated F(ab’)_2_:maleimide reaction ratio was at least 10:1 (and each F(ab’)_2_ contained 2 thiol groups). We have deferred measuring the actual number of F(ab’)_2_ grafted per bubble, because a robust methodology for this is currently lacking. However, with a 10 molar excess of F(ab’)_2_ relative to the maleimide functions, we expect the number of F(ab’)_2_ on a bubble to be roughly the same as the number of maleimide functions on the bubble, ie, ≈30k/μm^2^ bubble surface area or ≈400k/bubble. This assumes saturation of all maleimide functions, each linked to one F(ab’)_2_ molecule. Lower F(ab’)_2_:bubble reaction ratio of 1x10^6^:1 (≈2.5 molar excess of F(ab’)_2_ relative to the maleimide function) produced bubbles that could only attach to target under static conditions *in vitro* [2], presumably due to insufficient targeting ligand density achieved on the bubble-shell. The 10 molar excess of F(ab’)_2_ compares favorably with the 10 [20] or 30 [21, 22] molar excess employed by others in similar maleimide-thiol bubble conjugation reactions. BR55, a targeting bubble being tested for clinical translation by the Bracco pharmaceutical company [23, 24], used a 1:1 molar ratio in the conjugation reaction between its targeting ligand and free lipid containing the conjugation function, prior to bubble formation [25]. It is important to note that in general, ≤10% of the total shell components is incorporated into the bubble-shell (although the component molar ratio remains essentially unchanged) [26]. Therefore ≥90% of the targeting ligand is lost when using the pre-bubble conjugation strategy. Perhaps significantly the amount of targeting ligand lost is not dissimilar to the 10 molar excess used in the conjugation strategy reported here. Thus, the use of 10 molar excess targeting ligands relative to the maleimide function in the conjugation reaction should not make translation economically non-feasible. However, future studies would be desirable to: (1) determine the minimum excess of targeting ligands required to saturate all active (potentially toxic) maleimide functions on the bubbles; and (2) compare the measured number of targeting ligands bound per bubble with that predicted.

The use of F(ab’)_2_ instead of whole mAb as targeting ligands eliminated any Fc-medicated non-specific interaction or immunogenic side-effects. The translatability of a bubble may depend more on the bubble’s generic composition and conjugation chemistry than the targeting ligand *per se*, because the latter can be exchanged for one that is human-compatible and binds efficiently to the molecule of interest without significantly altering the bubble’s other properties important for clinical applications (eg, *in vivo* stability and acoustic response favourable for real-time and quantitative imaging; low degrees of non-specific binding/retention in multiple tissues favourable for broad applications; non-toxic/biocompatible). It remains to be determined whether or not the generic composition of our bubble (with minor modifications outlined in limitations below) can form the basis for generating a series of targeting bubbles against various molecules for potential clinical applications in the future.

Our bubbles were frozen for long-term storage. Common frozen products widely used in clinical practice include blood products such as the fresh frozen plasma and cryoprecipitate, and cells used in bone marrow transplantation or stem cell therapy. Although frozen formulations are feasible for clinical use, non-frozen formulations would be preferred for logistic reasons (easier transportation and long-term storage). Non-frozen bubble formulations include as a lyophilisate (dry powder) or aqueous lipid dispersion (solution) in a sealed vial containing the bubble gas, where the bubbles can be reconstituted by the bedside just prior to use [27–29]. However, they require extra steps (time consuming) and do not offer clear advantages in our early stage of bubble development. All three storage formulations are prone to generating fragments that compete against the bubbles for attachment to molecular targets. In the non-frozen formulations, some investigators re-wash the reconstituted bubbles before use [28], while others do not [30, 31]–washing inevitably leads to a degree of bubble loss. In our study, the once thawed targeting bubbles (without re-wash) worked well in practice. Their efficacy in ultrasound molecular imaging and quantification suggested no significant negative impact from the once freeze-thaw cycle or bubble fragments. Comparative studies to determine the amount of target competing fragments generated in the single freeze-thaw process used in the present study, as compared to bubble reconstitution from lyophilised powders (eg, BR55 used in Phase 0–2 clinical trials [23–25, 32]), effect on bubble targeting, and the threshold beyond which they would cause significant negative impact on molecular imaging is useful, and remains to be investigated.

Outside of our work, as far as we are aware from publications in the English language of phospholipid-shelled targeting microbubbles that have been tested *in vivo* for ultrasound molecular imaging (Table A in S1 File):

1. Eight bubbles based on maleimide-thiol conjugation chemistry have been reported. These bubbles differed from ours in terms of their generic composition, including the gas (eg, C_4_F_10_ [20, 22, 30, 31, 33–35] instead of C_3_F_8_), the use of maleimide derivatives (eg, MPB) [36, 37] instead of maleimide, or the number/type/combination/molar ratio of the shell components. Their general properties are therefore likely to differ from ours. Only three of the eight bubbles showed their effectiveness for ultrasound molecular imaging in more than one non-reticuloendothelial tissues [22, 30, 31, 34, 35, 38, 39]; none showed effectiveness for acoustic quantification of the targeted molecule to a high degree of quantification, Table A in S1 File.
2. One bubble was recently reported for ultrasound molecular imaging mono-specific for Esel, in ischemia-reperfusion injury of the heart [31], and in LPS-induced inflammation of the hind-limb muscle [33] in rats. However, unlike our bubble, it contained immunogenic streptavidin and its effectiveness for acoustic quantification of the targeted molecule to a high degree of quantification was not demonstrated, Table A in S1 File.
3. Four bubbles targeting selectins (not mono-specific for E-, P- or L-selectin) have been reported for the imaging of inflammation in the heart [31, 34, 40, 41], skeletal muscle [33], large bowel [30] or tumor [42] in animals. Unlike our targeting bubble which is mono-specific for Esel, they are not suitable for the specific detection of endothelial activation which starts early in inflammation. This is because P-selectin is expressed on both platelets and endothelial cells, while L-selectin is expressed on lymphocytes. Biotin-streptavidin linkage was used in three of these bubbles [33, 40–42], and maleimide-thiol linkage in the other [30, 31, 34] for grafting of targeting ligands to the bubbles, Table A in S1 File.

Elsewhere, ultrasound molecular imaging using targeting microbubbles mono-specific for Esel has recently been reported in ischemia-reperfusion injury of the heart in rats [43] and in tumors in mice [44]. The bubbles used in these studies differ to ours in several major aspects, eg, they are not phospholipid-shelled microbubbles and the conjugation chemistry used is different. One used a double-shelled albumin (outer shell)—poly-DL-lactide (inner shell) microbubble, grafted with targeting ligands using biotin-streptavidin conjugation chemistry [43]; the other used a poly n-butylcyanoacrylate-shelled microbubble grafted with targeting ligands using carbodiimide conjugation chemistry [44]. In both cases the bubbles effectiveness for real-time ultrasound molecular imaging and acoustic quantification of the targeted molecule were not demonstrated.

Esel is an endothelial adhesion molecule, classically expressed only on activated endothelial cells (basal expression is lacking and expression is absent in other cell types) [45]. It can therefore be used for the specific detection of endothelial activation, which occurs early in inflammation [46]. The classical vascular bed for Esel expression is the post-capillary venules [45]. However, it has also been found in the capillaries of the heart (in myocarditis) [47] and the glomeruli (in glomerulonephritis) [13] in mice, similar to our observations in LPS-induced inflammation of these tissues.

The mouse model of LPS-induced systemic inflammation was used in this study for imaging Esel expression in inflammation. Widespread Esel expression in multiple organs was produced, which allowed testing of our bubble for ultrasound molecular imaging in more than one tissue in the same animal in one setting. It is a recognised model of endotoxemia and inflammation; it reflects myocarditis or glomerulonephritis where widespread inflammation of the heart or kidney occurs, respectively [47, 48]. In contrast to the models of ischemia-reperfusion injury or transplant rejection, commonly used in this field for imaging inflammation, the LPS mouse model is relatively easy to generate as it does not require surgery. Furthermore, surgical trauma may confound analysis. The essentially global uniform expression of Esel in the heart in this model (and Esel being expressed only on activated endothelial cells) was advantageous for assessing the bubbles effectiveness for acoustic quantification of the targeted molecule, and for investigating the cause of localised signal attenuations in the heart. Future studies applying the bubble technology to other models of clinical disease would be desirable.

Ultrasound detection of Esel in the heart was limited by attenuations from the overlying bone/air and retained bubbles located proximally in the ultrasound path. However, such attenuations were overcome by using lower frequency ultrasound (greater penetrative depth) or different imaging plane/angle. From the human imaging perspective, where the use of lower frequency ultrasound (eg, 3–7MHz) and multi-plane imaging are the norm, and the footprint of the transducer is much smaller relative to the body size (making it easier to achieve optimal probe position/angle and avoid overlying bone/air), these attenuation issues are likely less important.

The *in vivo* specificity of our Esel targeting bubble was demonstrated using Esel KO mice as negative controls. The KOs were used appropriately for this purpose for several reasons. They were derived from the same genetic background and treated in the same way with inflammation inducing agent as the WT animals; its local shear against bubble attachment/retention was not significantly different from the WTs. Inflammation occurred in both the WT and KO animals, even though Esel was absent in the latter. Although the number of adherent and transmigrated leukocytes was higher in the WT animals [2], the retained bubbles were not seen attached or phagocytosed by the leukocytes, suggesting that bubble retention in the WT was essentially due to specific bubble attachment to Esel with little/no contribution from non-specific bubble-leukocyte interactions. Confocal microscopy further confirmed specificity of the bubble by demonstrating co-localization of the retained/attached bubbles with Esel expressed on the endothelial cell surface. The use of the KO mice as negative controls, in preference to using control bubbles conjugated with non-binding F(ab’)_2_, had the advantage of testing the Esel targeting bubbles themselves rather than inferring about them from testing of the control bubbles. It also avoided confounding due to differences in signal intensities from non-identical size distribution between the targeting and control bubbles. Furthermore, non-binding ligands on the control bubbles may well not be inert and hence may have their own contributions. An alternative negative control in which Esel is blocked with free targeting ligand of the bubble prior to bubble administration *in vivo* can be used. However this has its own limitations and was not deemed necessary given the extent of proof already obtained in this feasibility study.

### Limitations

In general, the bubble composition and processing conditions are important determinants of a bubble’s effectiveness for *in vivo* imaging applications, because they affect the bubble’s: (i) half-life (stability) and acoustic response (signal intensity), relevant for real-time and quantitative imaging (stable, echogenic, low attenuation bubbles with a relatively large linear range of bubble concentration *vs* signal intensity are preferred); and (ii) degree of non-specific binding/retention and toxicity (biocompatibility). These through their impact on the bubble size distribution, shell properties (eg, gas diffusivity, thickness, surface tension, viscoelasticity, charge and PEG content) and interactions with the host [2]. The mechanisms are not well understood and is an area of ongoing research beyond the scope of the present study.

The bubble dose used for ultrasound imaging was relatively large (10^8^ bubbles; 5x10^9^ bubbles/kg). This is approximately 10-fold the maximum dose of Sonovue (an FDA approved non-targeting bubble) tested in humans without causing adverse effects. The standard clinical dose for Sonovue (0.03ml/kg) is 0.006–0.015x10^9^ bubbles/kg (≈0.4-1x10^9^ bubbles for a 70kg man, assuming a bubble concentration of 2-5x10^8^/ml); the maximum dose tested was 56ml (1-3x10^10^ bubbles or ≈0.16–0.4x10^9^ bubbles/kg) [49]. There is no standard dose of targeting bubbles used in ultrasound molecular imaging. In mice, dosages used ranged 8x10^5^–2x10^8^ bubbles (≈0.03-8x10^9^ bubbles/kg), Table A in S1 File. Like other investigators [8, 50–52] we did not observe significant acute adverse effects using such a large dose of bubbles. Nonetheless, the safety of such dosage in humans is uncertain and caution is needed. Hu *et al* [53], using a dose of 10^8^ targeting bubbles in mice, reported that high power bubble destruction may induce transient reduction in blood flow in the targeted tissue region; the effect was reduced using lower ultrasound power. In our study, high power bubble destruction was not used. The purpose of the large dosage used in our study was two fold: (a) To provide excess bubbles to cover the full range of molecular expression being quantified in the heart, and account for bubble ‘steal’ by multiple tissues expression Esel. In man or other clinically relevant disease models, a smaller dose (relative to body weight) may be sufficient because the number of tissues and peak level of Esel expression are likely to be lower than that observed in the LPS mouse model used. We did not carry out a formal dose titration study to confirm that the bubble dose used was optimal, which would be useful as a future study. Nevertheless, the good correlation of *R24* with Esel throughout the expression range imaged suggested that the dosage used was adequate. (b) To allow more sensitivity detection of non-specific bubble retention. Our bubble appears to have one of the lowest degrees of non-specific retention compared with others. How much of this is due to differences in the disease model or vascular bed imaged, or ultrasound setting used is uncertain. Non-specific bubble retention may increase in the context of inflammation (due to increased leukocyte presence) and in low shear flow regions (due to increased chance of sustained bond formation and stasis eg, in the tumor neovasculature with blind ending loops). Different ultrasound settings (mode, power, gain) will have different sensitivities for bubble detection. We used low power CPS mode of a popular clinical ultrasound scanner for sensitive real-time bubble detection. In our hands, the bubble showed low degrees of non-specific retention in low shear flow regions of the heart, kidneys (and cremaster), in the context of inflammation and high dose bubble administration. Future studies targeting different disease processes, vascular beds and hosts will be required.

Formal immunogenicity and toxicity assessment of our targeting bubble was not performed. However, potentially toxic active (unreacted) maleimide groups remaining on the targeting bubble surface were minimised/eliminated by using excess targeting ligands in the bubble conjugation reaction. And potentially toxic residual thiols on the targeting ligands on the bubble were then inactivated using excess NEM, and unreacted NEM subsequently removed by wash-purification of the bubble. The potential immunogenicity/toxicity of the reacted maleimide on the bubble (including reacted NEM) was discussed previously. The resulting targeting bubble did not cause acute adverse effects in mice, but its medium- and long-term effects are unknown. Before testing in humans, minor modification of the bubble will be required (such as excluding DiI and using human-compatible targeting ligands) followed by formal immunogenicity and toxicity studies *in vitro* and in animals. To date, BR55, a VEGFR2 targeting bubble for cancer detection [54], different in composition and conjugation chemistry to ours (Table A in S1 File), is the only targeting bubble tested in humans (Phase-0-2 clinical trial in prostate cancer) [23, 24]–the results are still awaited. Notably, BR55 appeared to show moderate degrees of non-specific retention in the healthy tissue (rat prostate) [55].

## Conclusion

Targeting microbubbles for ultrasound molecular imaging, based on maleimide-thiol conjugation chemistry with the generic (non-targeting components) composition described, may possess properties (i)-(iii) desired for clinical applications.

## Supporting Information

S1 File (PDF)

S1 Video Intravital Microscopy of Esel targeting microbubbles in an IL-1β pre-treated WT mouse. Shortened movie clip containing CCD (bright field) and SIT (fluorescence) images. A vein and artery can be seen running in a 5 to 11 and 7 to 3 o’clock direction, respectively. Bubbles were administered via the tail vein at 14:30:15.
(MP4)

S2 Video Intravital Microscopy of Esel targeting microbubbles in an IL-1β pre-treated Esel KO mouse. Shortened movie clip containing CCD (bright field) and SIT (fluorescence) images. A vein and artery can be seen running in a 6 to 1 and 9 to 3 o’clock direction, respectively. Bubbles were administered via the tail vein at 12:58:35.
(MP4)
