# Supplementary material for: A Targeting Microbubble for Ultrasound Molecular Imaging
Source: PLoS One. 2015 Jul 10;10(7):e0129681. doi: 10.1371/journal.pone.0129681 (PMC4498921; doi:10.1371/journal.pone.0129681)
Supplement: S1 File — (PDF) [file pone.0129681.s001.pdf]

# Detailed Methods

## Antibodies

### 1. Labelling of MES-1 mAb with Alexa Fluor 488 (AF488) Fluorescence Dye for Confocal Microscopy.

MES-1 was labelled with Alexa Fluor 488 carboxylic acid, 2,3,5,6-tetrafluorophenyl ester, 5-isomer (Molecular Probes) as follows. A 100µL-labelling reaction mixture consisting of 50µmol/L MES-1 mAb (25.7µL at 194µmol/L (29.1mg/mL) in PBS pH 7.5), 750µmol/L AF488 (6.6µL at 11.3mmol/L (10mg/mL) in water), 2.6µL (1/10$^{th}$ volume of MES-1 mAb) 1mol/L NaHCO3 (pH 8.5) and 65.1µL distilled $H_2O$, was incubated at room temperature (rt) for 1h, with gentle manual agitation at 30min. Controls included no dye or no MES-1 mAb. AF488-labelled MES-1 was then purified from the reaction mixture and suspended in PBS (pH 7.5), using gel-filtration chromatography spin column with 6kDa size exclusion limit (Bio-Spin P-6 Column with SSC packing buffer, BioRad) according to the manufacturer's instructions with the following specific conditions: the Bio-Spin column was buffer exchanged first with PBS using 3 wash cycles; all centrifugations were carried out at 20°C. The concentration and degree of labelling of AF488-labelled MES-1 was determined using a spectrophotometer. Samples were diluted in PBS to 100µL volume in duplicates, and absorbance measured at 280nm ($A_{280}$) and 495nm ($A_{495}$). The concentration of the labelled mAb was calculated as: $[\text{mAb}]\,\text{in mg/mL} = \dfrac{A_{280} - (A_{495} \times 0.11)}{1.4} \times \text{dilution factor}$, where 0.11 is the correction factor for AF488's contribution to $A_{280}$. The concentration of the mAb in mg/mL was converted to µmol/L using: $[\text{mAb}]\,\text{in µmol/L} = \dfrac{[\text{mAb}]\,\text{in mg/mL}}{150000} \times 10^6$, where 150,000 is the molecular weight of

mAb in Da, $10^6$ is the multiplication factor for converting mol/L to μmol/L. The concentration of AF488 was calculated as: $[\text{AF488}]\,\text{in}\ \mu\text{mol/L} = \frac{A_{495}}{71000} \times \text{dilution factor} \times 10^6$, where 71,000 is the approximate molar extinction coefficient of AF488 dye (in $cm^{-1}M^{-1}$) at 494nm, $10^6$ is the multiplication factor for converting mol/L to μmol/L. Finally, the degree of labelling was calculated as: $\text{Degree of labelling} = \frac{[\text{AF488}]\,\text{in}\ \mu\text{mol/L}}{[\text{mAb}]\,\text{in}\ \mu\text{mol/L}}$. Aluminium foil was used at all stages to minimise light exposure. Under this protocol, unreacted AF488 was retained in the column, confirmed by the ‘no MES-1 mAb’ control reaction. The labelling reaction molar ratio of 15:1 (AF488:MES-1) yielded MES-1 labelled with 7 AF488 molecules each, labelling efficiency = 46%, yield ≈89%. The purified AF488-labelled-MES-1 (6.748mg/mL, 44.984μmol/L) was aliquoted, wrapped in aluminium foil and store at -20°C until use. The preservation of sensitivity and specificity to Esel of the AF488-labelled MES-1 was confirmed using immunohistochemistry on frozen heart sections of WT and Esel KO mice pre-treated with LPS.

**2. Reduction of MES-1 F(ab’)$_2$ for Microbubble Conjugation.** MES-1 F(ab’)$_2$ was reduced using 4 molar excess of tris(2-carboxyethyl)phosphine hydrochloride (Sigma-Aldrich): MES-1 F(ab’)$_2$ (83.3μmol/L, 8.3mg/mL) and tris(2-carboxyethyl)phosphine hydrochloride (333.3μmol/L, 0.096 mg/mL) in Exchange Buffer (50mmol/L 2-(N-Morpholino)ethanesulfonic acid (Sigma-Aldrich), 2mmol/L ethylenediaminetetraacetic acid (Sigma-Aldrich), pH 6) were incubated for 1h at 37°C under constant agitation. The reaction volume ranged 1-1.6mL. The reaction was stopped by placing on ice and immediate purification of the reduced F(ab’)$_2$ using spin column gel filtration chromatography with a 5mL-Zeba Desalt Spin Column (size exclusion limit 1,000Da) according to the manufacturer’s instructions (Perbio

Science), at 4°C. The spin column was previously equilibrated in cold Exchange Buffer. The degree of reduction of the $F(ab')_2$ (number of thiols per $F(ab')_2$) was determined spectrophotometrically using Ellman's test with Ellman's Reagent (Perbio Science) according to the manufacturer's instructions with the following modifications: the Ellman's reaction consisted of 2.5μL Ellman's Reagent (10mmol/L, 4mg/mL), 7.5μL Exchange Buffer and 90μL of the purified reduced $F(ab')_2$ in Exchange Buffer, incubated at rt covered with aluminium foil to minimise light exposure; absorbance at 412nm ($A_{412}$) was measured at 24min from the start of the Ellman's reaction, to determine the thiol concentration in the reduced $F(ab')_2$ sample by reference to a standard curve of Ellman's reaction with known concentrations of thiol-containing compound, L-Cysteine hydrochloride (Perbio Science) in Exchange Buffer (pH6); duplicate Ellman's 'blank' reaction where the Exchange Buffer was added in place of the reduced $F(ab')_2$ was used for baseline subtraction of $A_{412}$ from the test samples. The concentration of reduced $F(ab')_2$ was determined from $A_{280}$ in the absence of Ellman's Reagent (with the spectrophotometer zeroed using Exchange Buffer), as the latter would interfere with $A_{280}$. The degree of $F(ab')_2$ reduction was calculated as: $\text{thiol groups per F(ab')}_2 = \frac{[\text{thiol in μmol/L}]}{[\text{F(ab')}_2 \text{ in μmol/L}]}$. The reduced $F(ab')_2$ contained 2 thiol groups per molecule of $F(ab')_2$ – only 1 inter-chain disulfide bond was reduced per $F(ab')_2$. The purified reduced $F(ab')_2$ was kept at ≈80μmol/L (8mg/mL) in Exchange Buffer prior to conjugation with microbubbles. Note that the reduction of 1 inter-chain disulfide bond would not be sufficient to cause the dissociation of $F(ab')_2$ into 2 Fab' fragments, because $F(ab')_2$ of rat IgG2a contains 2 (not 1) inter-chain disulfide bonds linking the heavy chains together.

**Immunohistochemistry.** Immunohistochemistry was performed on acetone-fixed cryosections of freshly harvested hearts of WT (with/without LPS pre-treatment) and Esel KO (pre-treated with LPS) mice. After blocking non-specific binding sites with 100μL of 1:1000 rabbit serum (Sigma-Aldrich) for 1h at rt, sections were incubated for 1h at rt with 100μL of 0.067μmol/L (0.01mg/mL) primary antibody: MES-1 (for Esel), MEC13.3 (for PECAM-1, endothelial marker) or rat IgG2a,κ isotype negative control mAb. Each section was then incubated with 100μL of 0.034μmol/L (0.005mg/mL) biotinylated secondary antibody (biotinylated rabbit mAb against rat IgG2a) for 60min at rt. After blocking of endogenous peroxidase with 0.3% $H_2O_2$ methanol for 20-30min at rt, the horseradish peroxidase-based detection system, Vectastain ABC kit (Vector Laboratories), was used with 3,3′-Diaminobenzidine solution (SIGMA*FAST* DAB tablet, Sigma-Aldrich) as the chromogen substrate. Sections were counterstained using Harris Modified Hematoxylin Solution (Sigma-Aldrich) and 1% $NaHCO_3$, then dehydrated through 70-100% ethanol, dried and mounted with Histomount (VWR), and examined under light microscopy.

**RT-qPCR.** Freshly harvested tissues were kept in RNAlater solution (Ambion) to preserve RNA in-situ; total RNA was subsequently extracted using TRIzol reagent (Invitrogen) according to the manufacturer's instructions. The yield of total RNA from the mouse heart was typically ≈1μg pure RNA per 1mg tissue, kept at concentrations over ≈1mg/mL in molecular grade (RNase-free) $H_2O$ (Sigma-Aldrich). RT reaction for first-strand complementary DNA (cDNA) synthesis was performed using the Qiagen Omniscript Reverse Transcription kit (Qiagen) according to the manufacturer's instructions. The RT reaction mixture consisted of 1μg total RNA, 2μL 10x buffer RT, 2μL dNTP mix

(5mmol/L each dATP, dCTP, dGTP, dTTP), 1µL (4 units) Omniscript reverse transcriptase, 2µL (1µg) oligo(dT)$_{12-18}$ primer (Invitrogen) and molecular grade $H_2O$ made up to a total reaction volume of 20µL, incubated for 1h at 37°C. This was followed by real-time qPCR (SYBR Green detection method) for Esel and HPRT-I, according to the manufacturers' instructions. The primer sequences used were: Esel forward primer 5'-CTCATTGCTCTACTTGTTGATG-3', Esel reverse primer 5'-GCATTTGTGTTCCTGATTG-3', HPRT-I forward primer 5'-ATTAGCGATGATGAACCAG-3', HPRT-I reverse primer 5'-AGTCTTTCAGTCCTGTCCAT-3' (custom ordered from Invitrogen). The qPCR reaction was carried on a 96-well 0.2mL thin-wall PCR plate (Bio-Rad) covered with an Optical Quality Sealing Tape (Bio-Rad), using the iCycler$^{TM}$ (iCycler iQ Real-Time PCR Detection System, Bio-Rad) according to the manufacturer's instructions. The qPCR reaction volume was 25µL, consisting of 5µL cDNA template (1:50 water dilution of the finished RT reaction), 0.5µL (10µmol/L) each of the forward and reverse primer for the respective gene, 6.5µL molecular grade $H_2O$ and 12.5µL iQ SYBR Green Supermix (Bio-Rad). The qPCR cycling condition was: initial 3min denaturing step at 95°C (Well Factor analysis in the first 90s); then 40 cycles of 15s at 95°C, 1min at 56°C; melt-curve analysis in 0.5°C steps (1min denaturation at 95°C, 1min reset at 56°C, then 80 cycles of 10s at 60°C with 0.5°C increment for each cycle); final cooling step at 4°C. Triplicate Esel and HPRT-I were amplified on the same plate for each animal; no-template negative control using molecular grade $H_2O$ in place of cDNA template for both primer pairs were included in all plates. For data analysis, threshold cycle (*Ct*) was determined from the amplification plot using the iCycler iQ Optical System Software Version 3.0a (Bio-Rad). Wells with abnormal amplification plot or melt-curve were excluded. As PCR efficiency of the Esel and HPRT-I primer pairs differed by ≤5% (mean

(SD) = 93 (4%) and 92 (3%), respectively; n = 4 each), the comparative *Ct* method was used to estimate the amount of Esel mRNA relative to that of HPRT-I, using the formula: $\text{Esel mRNA (\%HPRT - I)} = 2^{-\Delta Ct}$, where $\Delta Ct = Ct_{\text{Esel}} - Ct_{\text{HPRT-I}}$, subscripts refer to the gene of interest. The mean of replicates was used.

**Microbubble Preparation.** A generic microbubble was prepared by dispersing DSPC, DSPE-PEG2000-Maleimide, PEG40-stearate and DiI at molar ratio of 75:9:14:2 in a small amount of cyclohexane:chloroform (1:2) solvent, in a 50mL round-bottomed flask. Excess solvent was extracted using a stream of gaseous nitrogen. The lipid-blend was then transferred to a freeze dryer and lyophilised to full dryness under a reduced atmosphere ($1.3\times10^4$ Pa) at -78.5°C (using a jacket of dry ice). The dry powder (lyophilisate) was then dispersed in normal saline containing propylene glycol (PGNS: propylene glycol 1.37mol/L (103.5mg/mL), glycerol 1.37mol/L (126.2mg/mL), NaCl 0.116mol/L (6.8mg/mL), pH ≈ 7.4) to a concentration of 4mg/mL, homogenised by sonication in an ultrasonic bath at 60-65°C until transparent. Once fully dissolved, the solution was gently sparged with $C_3F_8$-gas (F2 Chemicals). Microbubbles were then formed using a shear-mixing approach, by sonic dispersion of $C_3F_8$ using a Misonix 3000 sonicator (QSonica, CT). The probe tip was positioned about 2mm into the solution and sonication was performed with the high-intensity ultrasound horn (20-21 kHz) for 30-60s at an acoustic power of approximately 120W, with the initial temperature of the solution at ≈ 60°C. More $C_3F_8$-gas was sparged into the microbubble dispersion, and the vessel capped and immediately plunged into ice cold water (3min) to dissipate the heat generated during the sonication process. Microbubbles produced were washed (purified) by centrifugation flotation at

1,000g 4°C for 15-25min, using a Beckman Coulter Allegra X-15R Centrifuge (Beckman Coulter): bubbles float to the top of the sample vial after centrifugation, the subnatent was removed and replaced with equal volume of cold degassed normal saline (pH 7.4). The wash step was repeated 7 times to remove unincorporated shell components and bubble fragments. To produce Esel targeting microbubbles, these washed generic microbubbles were added to reduced MES-1 $F(ab')_2$ whilst mixing (each reduced $F(ab')_2$ molecule contained 2 thiol groups, prepared as described above). The conjugation reaction ratio was $4.338 x 10^6$ $F(ab')_2$ molecules per bubble (7.2nmol $F(ab')_2$ per $10^9$ bubbles). The total number of DSPE-PEG2000-Maleimide molecules used in the aqueous lipid-blend divided by the total number of bubbles (before wash) produced from the aqueous lipid-blend (measured by electrozone sensing as described in Methods) = $4.338 x 10^6$. If ≤10% of the components in the aqueous lipid-blend were incorporated into the bubble-shell, and the molar ratio of the components on the shell remained close to that in the lipid-blend [1], then a bubble of population mean size would contain ≤$4.338 x 10^5$ maleimide molecules, and the estimated $F(ab')_2$:maleimide conjugation reaction molar ratio would be ≥10:1. The concentration of bubbles and $F(ab')_2$ in the conjugation reaction mixture ranged 5-8x$10^9$/mL and 35-60μmol/L (3.5-6mg/mL), respectively. The reaction mixture contained approximately 2/3 volume of Exchange Buffer (pH 6) from the reduced $F(ab')_2$ and 1/3 volume of normal saline (pH 7.4) from the washed bubbles. The conjugation reaction was incubated at 4°C for 30min, continuously mixed gently on a vertically tilted rotating wheel. Bubble conjugation was terminated by adding 80mmol/L NEM dissolved in dry dimethyl sulfoxide (DMSO, Sigma-Aldrich) at 20 molar excess to $F(ab')_2$ - the reaction mixture was incubated at 4°C for 30-60min on the rotator. Typically, the concentration of NEM and DMSO in the reaction mixture was ≈1mmol/L

and ≤1.7% v/v, respectively. The bubbles were then washed 4 times with cold normal saline by centrifugation flotation as described above, at 160g 4°C for 5min. This removed unincorporated $F(ab')_2$, unreacted NEM, DMSO and bubble-fragments. To minimise bubble loss, all washes and incubations were performed with the bubble concentrations kept high (≥$1x10^9$ bubbles/mL), under $C_3F_8$ atmosphere (to reduce the concentration gradient for diffusion of $C_3F_8$-gas out of the bubbles) and at 4°C (to reduce the rate of gas diffusion). To preserve the bubbles DiI fluorescent dye, the lyophilisate or bubbles were protected from light. Freshly prepared washed Esel targeting bubbles were immediately divided into 20-50µL aliquots, capped and sealed with Parafilm, then snap frozen in liquid nitrogen and stored at -80°C until use. The concentrations of subsequently thawed Esel targeting bubbles ranged $1\text{-}3x10^9$ bubbles/mL amongst 5 batches prepared at different times (the range was due mainly to batch variation of the bubble concentrations in the conjugation and wash steps, rather than the freeze-thaw process – the latter caused only 14% change). The freeze-thaw strategy allowed long-term storage of the bubbles and minimised variations in the bubble size distribution (affecting eg, ultrasound signal intensities) used in the experiments. Thawed left over bubbles were not re-used.

**Targeting Microbubble Binding Assay *In Vitro*.** Polystyrene petri-dish (Corning 35mm Not TC-Treated Culture Dish, Corning Life Sciences) was coated with 200µL recombinant homodimeric mouse Esel protein (R&D Systems) at 1.25µg/mL (7nmol/L, diluted in PBS pH 7.5) for 1h at rt (dish E). PBS was used instead of Esel as 'blank' negative control (dish P). Non-specific binding sites were then blocked with 4mL bovine serum albumin (BSA, Sigma-Aldrich) at

2.5% w/v in PBS for 2h at rt. BSA was then discarded and the dish washed with PBS. To prepare Esel coated dish blocked with excess MES-1 F(ab')$_2$ (dish B), 500µL MES-1 F(ab')$_2$ at 6.67µg/mL (67nmol/L) was placed in dish E for 30min at rt, then washed with PBS. Due to the buoyancy of the bubbles, the dishes were inverted for incubation with bubbles for 1min at rt: 100µL Esel targeting or non-targeting (generic) bubbles at $2.5x10^7$ bubbles/mL (diluted in cold degassed PBS) were used. Unattached bubbles were then gently washed off and the dishes re-filled with cold degassed PBS for immediate examination using an upright light microscope equipped with immersion objective lens, connected to a camera and monitor. The number of bubbles attached to each dish was counted and averaged from 10 random OFs on the monitor display, the surface area of the latter determined using a stage micrometer. The attached bubble density was thus determined.

**Intravital Microscopy Set-Up.** Inflammation of the cremaster muscle was produced by intrascrotal injection of 50ng recombinant IL-1β (R&D Systems) 2h before surgery in the WT and Esel KO mice. The tail vein was cannulated with a 24G 0.7x19mm *iv* catheter (dead space ≈50µL) (BD Medical). Long duration anesthesia was achieved using *ip* injection of 200-300µL mixture containing 4.5mmol/L (1mg/mL) xylazine (Rompun, Bayer) and 36.5mmol/L (10mg/mL) ketamine hydrochloride (Ketalar, Parke-Davis) in normal saline. The animal was placed on a custom-built thermo-controlled (37°C) intravital microscopy stage. Under a dissection microscope, the right or left testis was gently exteriorised through a scrotal incision. A longitudinal incision was made along the cremaster muscle, which was then spread out and pinned down across a translucent microscopy stage. The exteriorised muscle was maintained by continuous super-fusion of thermo-controlled Tyrode's

Salt buffer solution (9.6g Tyrode's Salts (Sigma-Aldrich) + 1g sodium hydrogen carbonate, made up to 1L volume with sterile distilled water). Observations and recordings were made using an upright microscope (Axioskop, Carl Zeiss) equipped for bright-field and fluorescence microscopy, with 20x and 40x immersion objective lens (Water Achroplan, Carl Zeiss), a charge-coupled device camera (Color Chilled 3CCD Camera with controller, Hamamatsu Phototonics), a silicon intensifier target camera (C-2400-08, Hamamatsu Photonics), a monitor (Triton, Sony), a S-VHS recorder (Model AG 6730 SVHS 625, Panasonic) and a Personal Computer. All recordings were made with the S-VHS recorder and Personal Computer.

**Confocal Microscopy of Esel Targeting Microbubbles in the Mouse Cremaster.** Following the *iv* administration of Esel targeting bubbles and antibody cocktail containing AF488-MES-1 + allophycocyanin-labelled mAb against mouse PECAM-1, unattached bubbles and mAb in the circulation were removed by perfusion with PBS. This was achieved by exposing the heart and upper abdomen by dissection. A snip incision was then made in the right atrium followed by injection of PBS into the LV cavity using a needle connected to a 20ml-syringe. This allowed the PBS to perfuse the body, it and the blood left the circulation via the incision in the right atrium. Adequate PBS perfusion was assumed when the liver turned pale due to the replacement of blood by PBS. Then immediately, the testis were exteriorized and the cremaster muscles harvested, spread out and fixed in 4% paraformaldehyde PBS solution for 30min at rt, before placing in PBS at 4°C for 5min. Aluminium foil was used to minimise light exposure to the tissues. Fresh tissues were examined immediately under confocal microscopy (z-stacked), using an upright

confocal laser-scanning microscope (LSM 5 PASCAL, Carl Zeiss) with a 40x immersion objective lens (Water Achroplan, Carl Zeiss). 3 different fluorescence, DiI for bubbles (excite at 543nm, detect at 560-615nm), Alexa Fluor 488 for Esel (excite at 488nm, detect at 505-530nm) and allophycocyanin for PECAM-1 (excite at 633nm, detect at 650nm) were scanned in series at each depth before moving on to the next depth in the Z-axis. Images were processed using the Zeiss LSM 5 Image Browser.

**Ultrasound Imaging.** 15 WT and 8 Esel KO mice all pre-treated with LPS were imaged. Tail vein cannulation and general anesthesia were performed in the same way as described in the intravital microscopy experiment. The chest, abdomen and pelvis were shaved. The Acuson Sequoia 512 clinical ultrasound scanner equipped with a 15L8-s linear array transducer (foot print 26mm) was used. A layer of warm gel was coupled between the skin and ultrasound transducer. Ultrasound settings used were: 14MHz CPS mode, transmission power 9dB giving low MI 0.22-0.26 (estimated by the scanner), dynamic range 55dB, time gain 0%, CPS gain 8, fundamental 2D gain 15dB, color map M:3 (bubble signals in heated object scale ('CPS-contrast only' images), tissue signals in grey scale ('B-mode' images)). Tissue Equalization Technology (TEQ) was not used. Baseline images of the heart in the PSA (papillary muscle level), PLA and A4C views were acquired before bubble administration. Imaging was then maintained in the PSA view by fixing the transducer in position with a free standing clamp. A stopwatch was started and $10^8$ Esel targeting bubbles (in 100μL volume made up with normal saline) injected at 10s as an *iv* bolus over 1-2s through the tail vein cannula. This was followed by a 100μL normal saline flush over 1-2s at 20s. To capture real-time sequence of events throughout the life-time of the bubble bolus and detect Esel expression in the heart, continuous

ultrasound imaging was performed and recorded as 3s-digital clips, at pre-determined time intervals as follow. Continuous imaging was applied from time 0 to 1min 23s on the stopwatch, then for 3s each time at: (i) 1min intervals from 2min 20s to 10min 20s; (ii) 2min intervals from 12min 20s to 30min 20s; and (iii) 5min intervals from 35min20s to 60min 20s. 3s-digital clips for these were recorded at 10s, 13s, and then at 10s intervals from 20s to 1min 20s, then at: (i) 1min intervals from 2min 20s to 10min 20s; (ii) 2min intervals from 12min 20s to 30min 20s; and (iii) 5min intervals from 35min 20s to 60min 20s. Imaging was terminated earlier if bubble contrast enhancement in the LV cavity (central blood pool) was no longer visible. To determine the nature of bubble signal attenuation, the following was performed in addition: (i) other views of the heart (PLA, A4C) were acquired at the end; (ii) 7MHz CPS imaging at MI 0.22 (keeping the gain and other settings the same as 14MHz imaging) was acquired at baseline & end of the 14MHz imaging in some animals; and (iii) wider PSA view ('RES off') surrounding the heart was acquired at 5min intervals. When switching from 14MHz to 7MHz CPS imaging, the transmit power was first reduced from -9dB to -19dB before reducing the ultrasound frequency, to avoid an increase in MI (up to ≈0.7) causing inadvertent bubble destruction. To image extra-cardiac tissues, the thorax, abdomen and pelvis were imaged in the antero-posterior plane at baseline & end of the cardiac imaging study in some animals. To do this, the probe was positioned transversely and moved slowly caudal from just below the neck to the pelvis during image recording; 14MHz CPS images were acquired. All animals received only one dose of bubbles to eliminate any carry-over effects from previous bubble dosing (eg, blocking of Esel binding sites). The duration between LPS treatment and the administration of bubbles was noted as the $LPS_{Time}$. All animals were sacrificed at the end.

# Supplemental Figures and Figure Legends

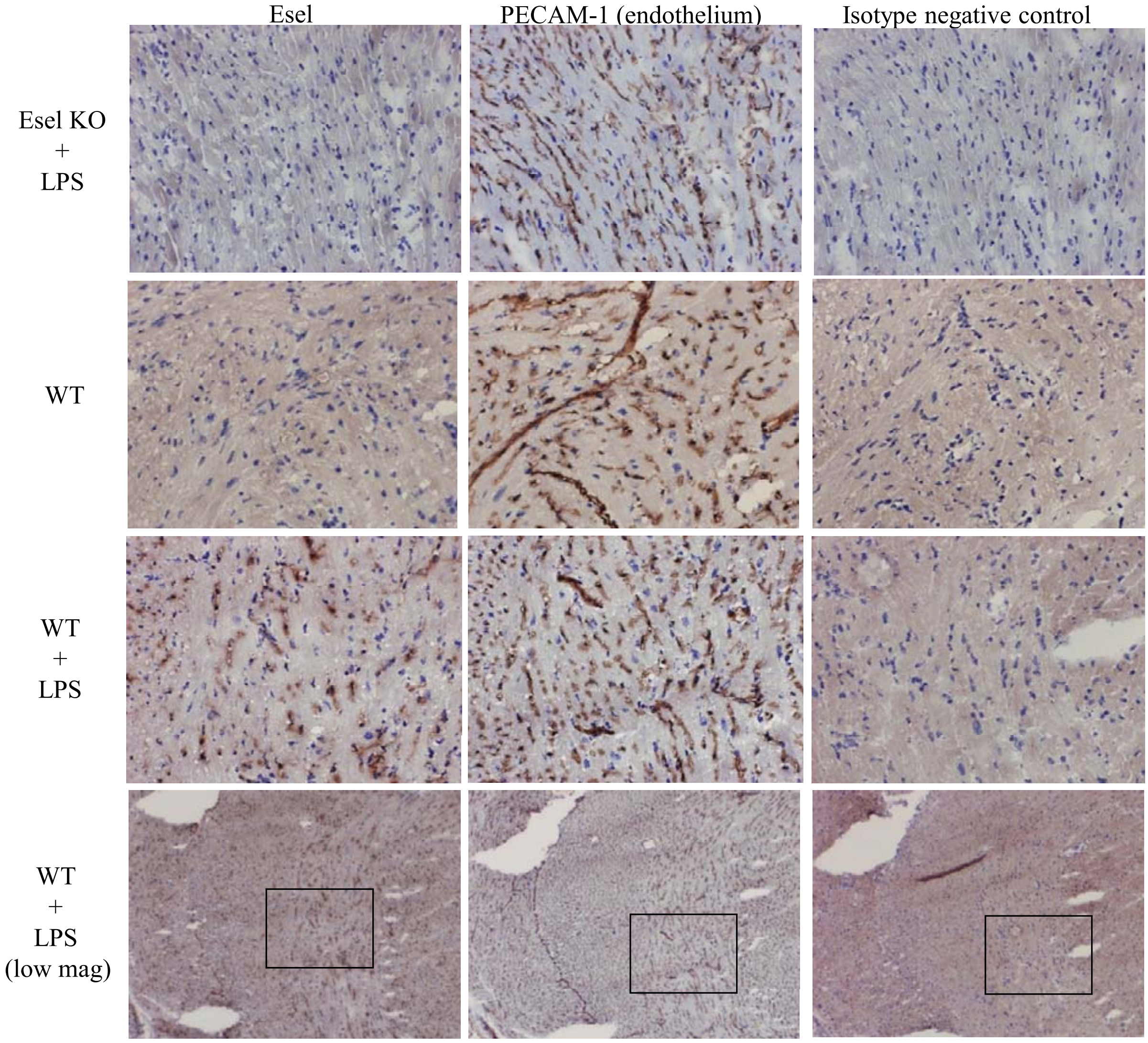


**Fig A. Frozen section immunohistochemistry of the heart.**

Representative examples from mice with/without LPS pre-treatment ≈6h beforehand. Positive staining = brown color. Magnification 200x. Low power magnification (low mag) 40x. Box = optical field for the 200x magnification in the WT mouse pre-treated with LPS.

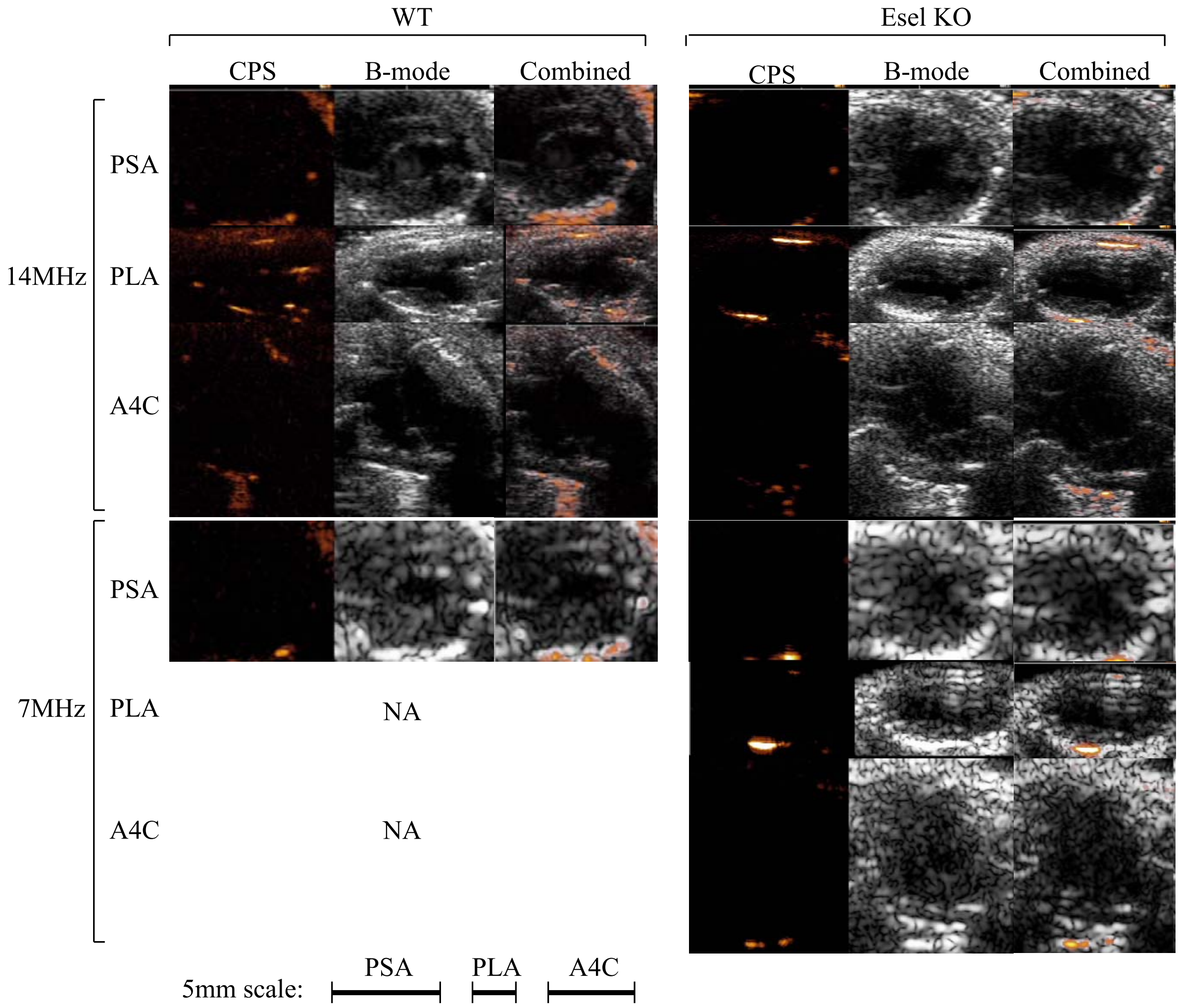


**Fig B. Baseline images (before bubble administration) for Fig. 6.**

| Origin<br><br>(Bubble ID, where available) | Conjugation Chemistry | Bubble Shell Composition | Bubble Gas | Targeting Ligand | Targeting Ligand Density on Bubble<br><br>*(Conjugation Reaction Ratio)* | Bubble Diameter (μm) | Targeted Molecules | Disease Model | Tissues Imaged | Host<br><br>&<br><br>Bubble Dose (number of bubbles) | Application[§§] | References |
|---|---|---|---|---|---|---|---|---|---|---|---|---|
| UVA | Biotin-streptavidin | • DSPC (83 mol%)<br>• PEG40-Stearate (16 mol%)<br>• DSPE-PEG2000-Biotin (1 mol%)<br>• ± DiI, DiO or rhodamine DHPE (small unspecified amount) | $C_4F_{10}$ | • mAb<br>• Peptide<br>• Protein (eg, YSPSL)[#] | 1.7-1.9k/μm$^2$<br><br>60k/ 3.2 or 3.4 μm bubble<br><br>*(Reaction ratio 1: 3M streptavidin molecule/ bubble; 3M mAb/ bubble (0.3-3M mAb/ bubble = saturating) → conjugated ratio unknown ( Lindner JR et al, 2001))*<br><br>*(Reaction ratio 2: 800k mAb/ bubble → conjugated ratio unknown ( Kaufmann BA et al, Arterioscler Thromb Vasc Biol* | Mean: 1.9-3.4<br><br>Range: 94-97% are <5 | • $\alpha_5$-integrins<br>• $\alpha_v$-integrins<br>• $\beta_1$-and $\beta_3$-integrins<br>• CX3CR-1<br>• ICAM-1<br>• VCAM-1<br>• MAdCAM-1<br>• P-selectin<br>• Platelet glycoprotein-1bα<br>• Selectins (not discriminating amongst E-, P-, and L-selectin)<br>• vWF (A1-domain) | • Angiogenesis (chronic ischemia, stem cell therapy, tumor)<br>• Coronary thrombosis & angioplasty<br>• Inflammation (atherosclerosis, cardiac transplant rejection, Crohn's disease (small bowel), ischemia-reperfusion injury) | • Aorta<br>• Bowel<br>• Heart<br>• Kidney<br>• Skeletal muscle<br>• Tumor (implanted in brain or flanks)<br>• Matrigel plug (implanted in abdomen) | Mouse: 1-10 x10$^6$<br><br>Rat: 2.5-100 x10$^6$<br><br>Dog: 10$^8$ | RT<br>SQ | • Lindner JR *et al*, Circulation 2001;104:2107<br>• Ellegala DB *et al*, Circulation 2003;108:336<br>• Leong-Poi H *et al*, Circulation 2003;107:455; Circulation 2005;111:3248<br>• Weller GE *et al*, Circulation 2003;108:218; Cancer Res 2005;65:533<br>• Sakuma T *et al*, Cardiovasc Res 2005;66:552<br>• Bachmann C *et al*, Gastroenterology 2006;130:8<br>• Villanueva FS *et al*, Circulation 2007;115:345<br>• Kaufmann BA *et al*, Circulation 2007;116:276; Eur Heart J 2007;28:2011; J Am Soc Echocardiogr 2010;23:79; Arterioscler Thromb Vasc Biol 2010;30:54<br>• Behm CZ *et al*, Circulation 2008;117:2902<br>• McCarty OJ *et al*, JACC Cardiovasc Imaging 2010;3:947<br>• Carr CL *et al*, Arterioscler Thromb Vasc Biol 2011;31:2526<br>• Davidson BP *et al*, J Am Coll Cardiol 2012;60:1690<br>• Khanicheh E *et al*, Arterioscler Thromb Vasc Biol 2013;33:2187 |

| | | | | | | | | | | | | |
|---|---|---|---|---|---|---|---|---|---|---|---|---|
| | | | | | *2010;30:54))* | | | | | | | • Liu Y *et al*, Circ Cardiovasc Imaging 2013;6:74<br>• Ryu JC *et al*, Circulation 2013;127:710 |
| UVA | Biotin-streptavidin | • DSPC (90 mol%)<br>• PEG40-Stearate (8.7 mol%)<br>• DSPE-PEG2000-Biotin (1.3 mol%)<br>• ± DiI, DiO (small unspecified amount) | $C_4F_{10}$ | • mAb | NA | Median: 2.03<br><br>Range: 1.5-6 | • H-2Kk (mouse MHC class I H-2Kk protein) on transfected bone-marrow derived endothelial progenitor cell | • Angiogenesis (stem cell therapy) | • Matrigel plug (implanted in abdomen) | Rat: $5x10^7$ | Not RT | • Kuliszewski MA *et al*, Cardiovasc Res 2009;83:653 |
| UVA | Biotin-streptavidin | • DSPC (72 mol%)<br>• PEG40-Stearate (28 mol%)<br>• DSPE-PEG3400-Biotin (<1 mol%)<br>• ± DiI (<1% mass of other lipids) | $C_4F_{10}$ | • Nanobody | 10k/$\mu m^2$<br><br>288k/ 2.2 µm bubble<br><br>*(Reaction ratio: 3M streptavidin molecule/ bubble; ≈3M nanobody/ bubble (saturating ratio))* | Mean: 2.2<br><br>Poly-dispersity index: 1.32 | • VCAM-1<br>• Enhanced green fluorescence protein (eGFP) | • Angiogenesis (tumor) | • Tumor (implanted in hind limb) | Mouse: 3.75 $x10^7$ | RT | • Hernot S *et al*, J Control Release 2012;158:346 |
| UVA | Biotin-streptavidin | • DSPC (83 mol%)<br>• PEG40-Stearate (16 mol%)<br>• DSPE-PEG3400-Biotin (1 mol%) | $C_4F_{10}$ | • mAb<br>• Peptide | NA | Mean: 3.2 | • Selectins (not discriminating between E- and P-selectin)<br>• VCAM-1<br>• VEGFR2 | • Angiogenesis (inflammation, tumor)<br>• Atherosclerosis | • Aorta<br>• Tumor (implanted in hind limb) | Mouse: 1-10 x $10^6$ | Not RT<br>SQ | • van Wamel A *et al*, Proc IEEE Ultrason Symp 2007;961 (abstract)<br>• Khanicheh E *et al*, PLos One 2013;8:e58761 |
| UVA<br><br>(Bubbles loaded with plasmid DNA) | Biotin-streptavidin | • DSPC (70 mol%)<br>• PEG40-Stearate (13 mol%)<br>• DSPE-PEG2000-Biotin (1 mol%)<br>• DSTAP (16 mol%)<br>• cDNA (plasmid containing firefly luciferase gene): 0.04pg loaded per bubble<br>• ±DiI or DiO | $C_4F_{10}$ | • mAb | NA | Mean: 2.5-3.1 | • P-selectin | • Microbubble targeted gene delivery in ischemia (ischemia-reperfusion) | • Skeletal muscle | Mouse: 2 $x10^8$ | Not RT | • Xie A *et al*, J Am Coll Cardiol Img 2012;5:1253 |

| | | | | | | | | | | | | |
|---|---|---|---|---|---|---|---|---|---|---|---|---|
| Targeson<br><br>(TargeStar-series) | Biotin-streptavidin | TargeStar/TargeStar-B<br>• biotin coated bubbles<br>• component details not disclosed<br><br>TargeStar-SA<br>• streptavidin coated bubbles, containing 2.7k streptavidin/μm$^2$ (streptavidin on distal tip of PEG)<br>• component details not disclosed | Perfluoro carbon (not specified) | • mAb<br>• Peptide<br>• scFvs | 8k/μm$^2$ or 100k scFv/ 2μm bubble (not actually measured) (Wang X *et al*, 2012)<br><br>*(Reaction ratio: 2.5M scFv /bubble: >20 (up to 25?) biotin/ streptavid-in binding site (each scFv has 1 biotin) → conjugated ratio 100k scFv/ 2μm bubble (not actually measured) (Wang X* et *al, 2012))* | Mean: 1.9-2.5<br><br>Range: 1-8; >98% are <8 | • $\alpha_v\beta_3$-integrin<br>• $\alpha_{IIb}\beta_3$ (GPIIb/IIIa)<br>• CD147*<br>• EGFR*<br>• P-selectin<br>• VEGFR2<br>• Hemagglutinin tag<br>• Single, dual or triple targeting of the following: $\alpha_v\beta_3$-integrin, P-selectin, VEGFR2 | • Angiogenesis (tumor)<br>• Thrombosis<br>• Tumorigene-sis | • Artery (carotid)<br>• Tumor (prostate)<br>• Tumor (implanted in flank, hind limb, kidney or mammary fat pad) | Mouse: 0.84-100x10$^6$ | RT<br>Q | • Rychak JJ *et al*, Mol Imaging 2007;6:289<br>• Xuan JW *et al*, Mol Imaging 2009;8:209<br>• Warram JM *et al*, J Ultrasound Med 2011;30:921<br>• Hu X *et al*, Invest Radiol 2012;47:398<br>• Sorace AG *et al*, J Ultrasound Med 2012;31:1543<br>• Knowles JA *et al*, Arch Otolaryngol Head Neck Surg 2012;138:662<br>• Wang X *et al*, Circulation 2012;125:3117<br>• Saini R *et al*, Ultrasound Med Biol 2013;39:172<br>• Wei S *et al*, Ultrasound Med Biol 2014;40:1250 |
| Targeson<br><br>(TS-02-008, bubbles loaded with plasmid DNA) | Biotin-streptavidin | • DSPC<br>• PEG40-Stearate<br>• DSPE-PEG40-Biotin (1%)<br>• DSTAP (2 mol%)<br>• cDNA (plasmid containing firefly luciferase gene): 0.04pg loaded bubble | $C_4F_{10}$ | • mAb | 20k/μm$^2$ or 200k/ bubble (not actually measured)<br><br>*(Reaction ratio*: *2M mAb/ bubble)* | Mean: 1.8 | • MAdCAM-1<br>• VCAM-1 | • Microbubble targeted gene delivery in Crohn's Disease | • Bowel | Mouse: 10$^7$ | RT | • Tlaxca JL *et al*, J Control Release 2013;165:216 |
| Visual-Sonics, Bracco<br><br>(Micro Marker Target | Biotin-streptavidin | • Streptavidin coated bubbles (streptavidin on distal tip of PEG)<br>• No further details disclosed | $C_4F_{10}$ + $N_2$ | • mAb<br>• Peptide | 6-8k/μm$^2$<br><br>40-54k/ 1.5μm bubble<br><br>*(Reaction* | Mean: 1-3 | • $\alpha_v\beta_3$-integrin<br>• Human CD276 on transfected mouse vascular endothelial cell<br>• DEspR<br>• Endogolin | • Angiogenesis (atherosclerosis, tumor)<br>• Inflammation (atherosclerosis, ischemia-reperfusion | • Artery (carotid, femoral)<br>• Bowel<br>• Brain (mouse embryo) | Mouse: 3.8-7.5 x10$^7$<br><br>Mouse embryo: 10$^5$<br><br>Rat: | RT<br>Q | • Bettinger T *et al*, Proceedings of the 12$^{th}$ European Symposium on Ultrasound Contrast Imaging 2007;81 (Rottedam) (abstract)<br>• Lyshchik A *et al*, J Ultrasound Med |

| | | | | | | | | | | | | |
|---|---|---|---|---|---|---|---|---|---|---|---|---|
| Ready Contrast Agent) | | | | | *ratio 1: 140k mAb/ bubble; 2.4 mAb/ streptavid-in molecule → conjugated ratio 40-50k/ 1.5µm bubble (not actually measured) (Willmann JK et al, Radiology 2008;246:508))*<br><br>*(Reaction ratio 2: 400k mAb/ bubble → conjugated ratio 54k mAb/ 1.5µm bubble (measured) (Deshpande et al, 2011))*<br><br>*(Reaction ratio 3: 500k mAb/ bubble → conjugated ratio unknown (Foygel K et al, 2013))* | | • ICAM-1<br>• P-selectin<br>• Thy1<br>• VCAM-1<br>• VEGFR2 | injury, TNFα - induced)<br>• Mural haemorrhage | • Hind limb<br>• Kidney<br>• Tumor (thyroid, pancreas)<br>• Tumor (implanted in flank, hind limb, mammary fat pad, pancreas or prostate)<br>• Vasa vasorum neovessel (carotid) | 3.1 ($1.3x10^8$/kg)- 40 $x10^7$<br><br>Rabbit<br><br>Monkey: $10^8$ | | 2007;26:1575<br>• Lee DJ *et al*, J Ultrasound Med 2008;27:855<br>• Willmann JK *et al*, Radiology 2008;246:508, Radiology 2008;248:936, J Nuc Med 2010;51:433<br>• Andonian S *et al*, J Endourol 2009;23:373<br>• Lee SC *et al*, JACC Cardiovasc Imaging 2010;3:1265<br>• Tardy I *et al*, Invest Radiol 2010;45:573<br>• Decano JL *et al*, Mol Imaging Biol 2011;13:1096<br>• Deshpande N *et al*, Radiology 2011;258:804; Radiology 2012;262:172<br>• Foygel K *et al*, Gastroenterology 2013;145:885<br>• Mancini M *et al*, BMC Medical Imaging 2013;13:31<br>• Lutz AM *et al*, Clin Cancer Res 2014;20:1313<br>• Chadderdon SM *et al*, Circ 2014;129:471<br>• Rix A *et al*, Ultrasound Med Biol 2014;40:2468<br>• Denbeigh JM *et al*, Ultrasound Med Biol 2014;40:389 |

| | | | | | | | | | | | | |
|---|---|---|---|---|---|---|---|---|---|---|---|---|
| SMU | Biotin-streptavidin | ND | ND | • mAb | NA | NA | • P-selectin | • Ischemia-reperfusion injury | • Kidney | Mouse | RT(NS) Q | • Bin JP et al, Eur Heart J 2008;29(Suppl 1):21 (abstract) |
| SMU | Biotin-streptavidin | Unspecified modification of bubbles from UVA | $C_3F_8$ | • mAb | NA | Mean: 2.4 | • $\alpha_v$-integrin<br>• ICAM-1 | • Angiogenesis (ischemia)<br>• Ischemia-reperfusion injury | • Heart<br>• Hind limb | Mouse: 5-10 x$10^6$ | RT SQ | • Yan Y *et al*, Cardiovasc Res 2011;89:175<br>• Xie J *et al*, Cardiovasc Res 2011;92:256 |
| SMU† | Biotin-streptavidin | • DPPC (55 mol%)<br>• PEG40 Stearate (20 mol%)<br>• DSPE-PEG2000-Biotin (4 mol%)<br>• ±DiI (21 mol%) | $C_3F_8$ | • mAb | Streptavidin mAb:bubble ≈ $10^5$:3x$10^5$:1 or 18k/μm$^2$ (method unknown)<br><br>*(Reaction ratio: 1M mAb/ bubble*) | Mean: 2.3<br><br>IQR: 1.7-3.6 | • VCAM-1 | • Atherosclerosis | • Artery (abdominal aorta) | Mouse: $10^6$ | RT SQ | • Wu J *et al*, Radiology 2011;260:463 |
| UCA Davis | Biotin-NeutrAvidin | • DSPC (90 mol %)<br>• DSPE-PEG2000 (5 mol %)<br>• DSPE-PEG2000-Biotin (5 mol %) | $C_4F_{10}$ | • Peptide | 0.6k/μm$^2$<br><br>3 or 7k/ 1.3 or 1.8μm bubble, respectively | Mode: 1.8, 4.5, 7.5<br><br>Median: 1.3 | • $\beta_1$-and $\beta_3$-integrins | • Angiogenesis | • Matrigel plug (implanted in groin) | Rat: 4x$10^8$ | RT | • Stieger SM *et al*, Contrast Media Mol Imaging 2008;3:9 |
| UCA Davis | Biotin-avidin | ND | ND | • Peptide | NA | Mean: ≈2 | • $\alpha_v\beta_3$-integrin | • Angiogenesis (tumor) | • Tumor (implanted in mammary fat pad) | Mouse: $10^8$ | RT | • Hu X *et al*, Invest Radiol 2012;47:398 |
| UNC | Biotin-streptavidin | • DSPC (90 mol%)<br>• PE-PEG2000 (5 mol%)<br>• PE-PEG2000- Biotin (5 mol%) | $C_4F_{10}$ | • pAb | NA | NA | • Secreted frizzled related protein 2 | • Angiogenesis (tumor) | • Tumor (implanted in hind limb) | Mouse: 5x$10^6$ | RT | • Tsuruta JK *et al*, PLosOne 2014;9:e86642 |
| WKU | Biotin-avidin | • DSPC (77 mol%)<br>• PEG40-Stearate (15 mol%)<br>• DSPE-PEG2000-Biotin (8 mol%) | $C_4F_{10}$ | • Peptide | NA | Mean: 3.2‡ | • $\alpha_v\beta_3$-integrin | • Angiogenesis (tumor) | • Tumor (implanted in inguinal area) | Mouse: $10^9$/kg (2.25x$10^7$) | RT | • Jun HY *et al*, Acad Radiol 2010;17:54 |
| TMMU | Biotin-streptavidin | • DPPG<br>• DSPC | $C_3F_8$ | • mAb | *(Reaction ratio:* | NA | • VEGFR2 | • Atherosclerosis (neovascularisa | • Artery (abdominal | Rabbit | RT(NS) SQ | • Liu H *et al*, J Clin Ultrasound 2011;39:83 |

| | | | | | | | | | | | | |
|---|---|---|---|---|---|---|---|---|---|---|---|---|
| | | • DSPE-PEG2000-Biotin<br>• PEG4000 | | | *≤12M mAb /bubble; ≤4 mAb/ streptavid-in molecule)* | | | tion) | aorta) | | | |
| NTHU<br><br>(Bubbles loaded with drug) | Biotin-avidin | • DPPC (66 mol%)<br>• DSPE-PEG2000 (17 mol%)<br>• DSPE-PEG2000-Biotin (17 mol%)<br>• BCNU (2.8mg)§ | $C_3F_8$ | • mAb | 9k/μm$^2$<br><br>92k/1.8μm bubble | Mean: 1.8 | • VEGFR2 | • Angiogenesis (tumor) | • Tumor (implanted in brain) | Rat: 6.1 x10$^9$ | RT(NS) | • Fan CH *et al*, Biomaterials 2013;34:2142 |
| Bracco (BG0470) | Maleimide - thiol | • DPPE-MPB (5 mol%)<br>• Other components not disclosed | ND | • Avidin$^{||}$ | NA | NA | • αIIbβ3 (GPIIb/IIIa) | • Thrombosis | • Artery (abdominal artery thrombus) | Rabbit | RT | • Tardy I et al, Acad Radiol 2002;9(Suppl 2):S294 |
| Bracco | Maleimide - thiol | • DSPC (37.5 mol%)<br>• DPPG (37.5 mol%)<br>• Palmitic acid (20 mol%)<br>• DPPE-MPB (5 mol%) | $C_4F_{10}$ | • Fab | 2k/μm$^2$<br><br>17k/bubble | Mean: 1.5-3.2<br><br>Range: >95% are <8 | • αIIbβ3 (GPIIb/IIIa) | • Thrombosis | • Artery (human blood in rat carotid) | Rat | RT | • Alonso A et al, Stroke 2007;38:1508<br><br>(related publications without *in vivo* studies: Martin MJ et al, Stroke 2007;38:2726. Della Martina A et al, Eur J Pharm Biopharm 2008;68:555) |
| Bracco | Maleimide – thiol-streptavidin-biotin | • DSPC<br>• Palmitic acid<br>• DSPE-PEG2000-Maleimide (5 mol%)<br>• Thiolated streptavidin coupled to maleimide on the bubbles (each bubble contained 22,000 molecules of streptavidin per μm$^2$) | $C_4F_{10}$ + $N_2$ (35:65 v/v) | • mAb<br>• Peptide<br>• Protein (YSPSL)$^{\#}$ | 3-86k/ μm$^2$<br><br>21-608k/ 1.5μm bubble<br><br>*(Reaction ratio 1: 40k mAb or YSPSL /bubble; 0.6 biotin/ streptavid-in molecule; 0.15 biotin/ streptavid-in binding site (each mAb or YSPSL has* | NA | • E-selectin<br>• P-selectin<br>• Selectins (not discriminating between E- and P-selectin) | • Inflammation (ischemia-reperfusion injury, LPS-induced) | • Heart<br>• Skeletal muscle (hind-limb) | Rat:<br>1-5 x10$^8$/kg<br>(3.3-15 x10$^7$) | RT<br>SQ | • Bettinger T *et al*, Invest Radiol 2012;47:516<br>• Hyvelin JM *et al*, Invest Radiol 2014;49:224 |

| | | | | | | | | | | | | |
|---|---|---|---|---|---|---|---|---|---|---|---|---|
| | | | | | *2-3 biotin) → conjugated ratio 21k mAb or YSPSL/ 1.5µm bubble (measured) (Bettinger T et al, 2012))*<br><br>*(Reaction ratio 2: 1M peptide /bubble; 16 biotin/ streptavid-in molecule; 4 biotin/ streptavid-in binding site (each mAb or YSPSL has 2-3 biotin) → conjugated ratio 608k/ 1.5µm bubble (measured) (Bettinger T et al, 2012))* | | | | | | | |
| | Maleimide – thiol | • DSPC<br>• Palmitic acid<br>• DSPE-PEG2000-Maleimide (5 mol%) | $C_4F_{10}$ + $N_2$ (35:65 v/v) | • Protein (YSPSL)# | 3k/µm$^2$ or 21k/1.5µm bubble (method unknown) | Mean: 1.5<br><br>Range: 1–2 | • Selectins (not discriminating between E- and P-selectin) | • Inflammation (acute colitis, ischemia-reperfusion injury) | • Heart<br>• Large bowel | Mouse: 5 x10$^7$<br><br>Rat: 2.6 x10$^8$/kg (8.7x10$^7$)<br><br>Monkey: 2 x10$^8$ | RT SQ | • Wang H *et al*, Radiology 2013;267:818<br>• Hyvelin JM *et al*, Invest Radiol 2014;49:224<br>• Davidson BP *et al*, J Am Soc Echocardiogr 2014;27:786 |

| | | | | | | | | | | | | |
|---|---|---|---|---|---|---|---|---|---|---|---|---|
| Imperial College London[##] | Maleimide - thiol | • DSPC (75 mol%)<br>• PEG40-Stearate (14 mol%)<br>• DSPE-PEG2000-Maleimide (9 mol%)<br>• DiI (2 mol%) | $C_3F_8$ | • $F(ab')_2$<br><br>(each reduced $F(ab')_2$ contained 2 thiol groups for maleimide conjuga-tion) | 28.5k/µm$^2$ or 434k/ 2.2µm bubble (not actually measured)<br><br>*(Reaction ratio ≈ 4M $F(ab')_2$ / bubble; ≥10$F(ab')_2$ /maleimide (each $F(ab')_2$ has 2 thiols))* | Mean: 2.2<br><br>Range: 98.6% are <6; 100% are <10 | • E-selectin | • Inflammation (LPS-induced) | • Heart<br>• Kidney | Mouse: $10^8$ | RT<br>Q | • Yeh JSM *et al*, J Am Coll Cardiol 2008;51(Suppl): A124-5 (abstract)<br>• Yeh JSM *et al*, Heart 2008; 94(Suppl II);A21 (abstract)<br>• Yeh JSM *et al*, Eur Heart J 2008;29(Suppl 1):21 (abstract)<br>• Current manuscript |
| UVA/ Targeson | Maleimide - thiol | • PC (which?)<br>• PEG40-Stearate<br>• DSPE-PEG2000-Maleimide | $C_4F_{10}$ | • Peptide | 6k/µm$^2$<br><br>120k/ bubble<br><br>*(Reaction ratio: 10 peptide/ maleimide (each peptide has 2 thiols))* | Mean: 2.5<br><br>Range: >99% are <8 | • VEGFR2 | • Angiogenesis (tumor) | • Tumor (implanted in hind limb) | Mouse: 2 x$10^7$ | RT | • Anderson CR *et al*, Invest Radiol 2010;45:579 |
| SMU | Maleimide – thiol (of lipid-peptide before bubble formation) | • DPPC (97.2 mol%)<br>• Poloxamer-188 (1.8 mol%)<br>• DSPE-PEG3400-Maleimide-Peptide (1 mol%)<br><br>DSPE-PEG3400-Maleimide-Peptide generated using conjugation reaction ratio: 1 peptide/30 DSPE-PEG3400-Maleimide (Hu G *et al*, 2012)? | $C_3F_8$ | • Peptide | 5-7k/um$^2$<br><br>110k/ bubble | Mean: 2.3-2.5 | • $\alpha_{IIb}\beta_3$ (GPIIb/IIIa) | • Thrombosis | • Artery (thrombus in abdominal aorta or carotid) | Mouse: $10^6$<br><br>Rat: $10^6$ | RT<br>SQ | • Hu G *et al*, Thromb Haemost 2012;107:172<br>• Wu W *et al*, Invest Radiol 2013;48:803 |

| | | | | | | | | | | | | |
|---|---|---|---|---|---|---|---|---|---|---|---|---|
| Borden[##] | Maleimide - thiol | • DSPC (90 mol%)<br>• DSPE-PEG2000 (5 mol%)<br>• DSPE-PEG2000-Maleimide (5 mol%) | $C_4F_{10}$ | • Peptide | *(Reaction ratio: 30 peptide/ maleimide)* | Size selected to 4-5µm<br><br>(actual measure-ments not given) | • $\alpha_v\beta_3$-integrin | • Angiogenesis (tumor) | • Tumor (implanted in kidney or hind limb) | Mouse: 2.5 x$10^7$<br><br>Rat: 5 x$10^6$ | RT<br>SQ | • Sirsi SR *et al*, Ultrasound Med Biol 2012;38:1019<br>• Borden MA *et al*, Mol Imaging 2013;12:357 |
| Borden[##] | Maleimide - thiol | • DSPC (90 mol%)<br>• DSPE-PEG5000 (5 mol%)<br>• DSPE-PEG2000-Maleimide (5 mol%) | $C_4F_{10}$ | • Peptide | *(Reaction ratio: 30 peptide/ maleimide)* | Size selected to 4-5µm<br><br>(actual measure-ments not given) | • $\alpha_v\beta_3$-integrin | • Angiogenesis (tumor) | • Tumor (implanted in hind limb) | Rat: 5 x$10^6$ | RT | • Borden MA *et al*, Mol Imaging 2013;12:357 |
| UT<br><br>(Bubbles loaded with plasmid DNA) | Maleimide - thiol | • HSPC (96 mol%)<br>• DOTMA (6 mol%)<br>• DSPE-PEG2000-Maleimide (2 mol%)<br>• pcDNA3 plasmids containing either the firefly luciferase or mouse Timp3 gene (0.20 mg/kg body weight incubated with 0.10 mL bubbles) | $C_3F_8$? | • mAb | *(Reaction ratio: 30mAb/ maleimide → conjugated ratio unknown)* | Mean: 2.1 | • MMP2 (extravascular target)** | • Microbubble targeted gene delivery in myocardial infarction (ischemia – reperfusion) | • Heart | Rat: 0.2ml (imaging)<br><br>0.1ml (therapy)<br><br>Bubble concentra-tion not disclosed | RT? | • Yan P *et al*, Biomaterials 2014;35:1063 |
| ImaRx Therapeu-tics (MRX-408) | Succinate-amine reaction forming carboxamide linkage (of lipid-peptide before bubble formation) | • DPPG-PEG- Peptide in Aerosome (MRX-113, ImaRx) lipid mixture<br>• No further details disclosed | $C_4F_{10}$ | • Peptide | NA | Mean: 2-2.5 | • αIIbβ3 (GPIIb/IIIa) | • Thrombosis | • Vein (inferior vena cava or left atrial appendage thrombus) | Dog: 6 x$10^7$/min | Not RT | • Takeuchi M et al, J Am Soc Echocardiog 1999;12:1015 |
| Bracco (BG0024) | Undisclosed covalent conjugation chemistry (of lipid- | • Lipid (which?) - peptide<br>• Other components not disclosed | ND | • Peptide | NA | NA | • $\alpha_{IIb}\beta_3$ (GPIIb/IIIa) | • Thrombosis | • Artery (abdominal artery thrombus) | Rabbit | RT | • Tardy I *et al*, Acad Radiol 2002;9(Suppl 2):S294 |

| | | | | | | | | | | | |
|---|---|---|---|---|---|---|---|---|---|---|---|
| | peptide before bubble formation) | | | | | | | | | | |
| Bracco (BR55; BG5075) | Amine-succinimidyl linkage[††] (of lipid- peptide before bubble formation) | • DSPE-PEG2000 & DSPE-PEG2000 - Peptide (at 15:1 mol:mol?)<br>• Other components not disclosed<br><br>DSPE-PEG2000 - Peptide generated using conjugation reaction ratio:<br>1 peptide/ DSPE-PEG2000; yield = 62% (Pillai R *et al*, 2010) | $C_4F_{10}$ + $N_2$ | • Peptide | 34±1k, range 32-37k or $57k/\mu m^2$<br><br>240k/ 1.5μm bubble or 400k/bubble | Mean: 1.5<br><br>Range: 1-3, or 97% are <4 | • VEGFR2 | • Angiogenesis (tumor)<br>• Normal liver | • Tumor (breast, liver, pancreas or prostate)<br>• Tumor (implant in flank, hind limb, mammary fat pad or prostate) | Mouse: 0.2-10 x$10^7$<br><br>Rat: 0.94-3.7x$10^7$; 0.1ml/kg (1.6μL bubble volume/kg)<br><br>Man (Phase 0-2 clinical trial) | RT<br>Q | • Pochon S *et al*, Invest Radiol 2010;45:89<br>• Tardy I *et al*, Invest Radiol 2010;45:573<br>• Fischer T *et al*, Invest Radiol 2010;45:675<br>• Pillai R *et al*, Bioconj Chem 2010;21:556<br>• Pysz MA *et al*, Radiology 2010;256:519, Quant Imaging Med Surg 2012;2:68, Radiology 2014 Oct 14 (Epub ahead of print)<br>• Bzyl J *et al*, Eur Radiol 2011;21:1988, Eur Radiol 2013;23:468<br>• Frinking PJ *et al*, Ultrasound Med Biol 2012;38:1460<br>• Sugimoto K *et al*, J Ultrasound Med 2012;31:1909<br>• Bachawal SV *et al*, Cancer Res 2013;73:1689<br>• Grouls C *et al*, Radiology 2013;267:487<br>• Wijkstra H *et al*, Proceedings of the 18th European Symposium on Ultrasound Contrast Imaging 2013 (Rotterdam)<br>• Wang H *et al*, Invest Radiol 2015 Jan 8 (Epub ahead of print)<br>• ClinicalTrials.gov, National Institutes of Health, NCT01253213 (2010-12) and NCT02142608 (2014-15) |
| XJU | Benzotriazole-amine reaction forming carbamate linkage | • DPPG (73 wt%)<br>• DSPE-PEG-BTC (21 wt%)<br>• DPPG:DSPE-PEG-BTC-Peptide | $SF_6$ | • Peptide | ? | Mean: ≈4.4<br><br>Range: >90% are | • $\alpha_{IIb}\beta_3$ (GPIIb/IIIa) | • Thrombosis | • Vein (femoral vein thrombus) | Dog | RT(NS) | • Wang B *et al*, Acad Radiol 2006;13:428 |

| | | | | | | | | | | | | |
|---|---|---|---|---|---|---|---|---|---|---|---|---|
| | (of lipid-peptide before bubble formation) | (6 wt%) | | | | 1-10 | | | | | | |
| UNC | Undisclosed covalent conjugation chemistry (of lipid-peptide before bubble formation) | • DSPC (90 mol%)<br>• DSPE-PEG2000 (5 mol%)<br>• DSPE-PEG2000-Peptide (5 mol%) | ND | • Peptide | NA | NA | • $\alpha_v\beta_3$-integrin | • Angiogenesis (tumor) | • Tumor (implanted in flank) | Rat: 1.4x$10^8$ | RT SQ? | • Streeter JE *et al*, Mol Imaging 2010;9:87, Mol Imaging 2011;10:460, Technol Cancer Res Treat 2013;12:311<br>• Gessner RC *et al*, Ultrasound Med Biol 2012;38:651 |
| Targeson (Visistar-Integrin) | Pyridyl Disulfide- -thiol | • DSPC<br>• PEG40-Stearate<br>• DSPE-PEG-PDP<br>• ± DiI (2 mol%) | $C_4F_{10}$ | • Peptide | 35k/$\mu m^2$<br><br>820±160k/ bubble<br><br>*(Reaction ratio: 5 peptide/ PDP (each peptide has 1 thiol) (Anderson CR et al, 2011))* | Mean: 2.75<br><br>Range: >98% are <8 | • $\alpha_v\beta_3$-integrin | • Angiogenesis (tumor) | • Tumor (implanted in mammary fat pad) | Mouse: 5-10 x $10^7$ | RT | • Anderson CR *et al*, Invest Radiol 2011:46:215<br>• Hu X *et al*, Am J Nucl Med Mol Imaging 2013;3:336 |
| UCA La Jolla /UC‡‡## | DNA-DNA linkage (non-covalent) | • DPPC 18mg + DPPA 2mg (mixture at 1mg/mL)<br>• DSPE-PEG5000 (0.075mg/mL)<br>• DSPE-PAA-DNA (mean 3.3 DNA strands per DSPE-PAA molecule) (0.3mg/mL) | $C_4F_{10}$ | • Aptamer (TACS) | *(TACS added to bubbles at molar ratio 1.5:1 relative to the available number of DNA sites)* | Mean: 1.6 | • Thrombin | • Thrombosis (active clotting) | • Vein (vena cava thrombus) | Rabbit: $10^7$ | RT | • Nakatsuka MA *et al*, Biomaterials 2013;34:9559 |

**Table A. Phospholipid-Shelled Targeting Microbubbles Tested For Ultrasound Molecular Imaging *In Vivo*.** Targeting microbubbles based on phospholipid-shell and published in the English language up to 7th March 2015 (PubMed search). Targeting ligands are conjugated to the microbubbles after they are formed, unless indicated otherwise. **Abbreviations:** abstr (abstract), BCNU (1,3-bis(2-chloroethyl)-1-nitrosourea, also known as Carmustine – an anti-neoplastic agent against eg malignant glioma), Conc (concentration), DEspR (dual endothelin-1/vascular endothelial growth factor-signal peptide receptor), DHPE (dihexadecanoyl phosphoethanolamine), DiI (1,1'-dioctadecyl-3,3,3',3'-tetramethylindocarbocyanine perchlorate), DiO (3,3'-dioctadecyloxacarbocyanine perchlorate), DOTMA (1,2-di-O-octadecenyl-3-trimethylammonium propane), DPPC (1,2-dipalmitoyl-*sn*-glycero-3-phosphatidylcholine), DPPE-MPB (1,2-dipalmitoyl-*sn*-glycero-3-phosphoethanolamine-4(p-maleimidophenyl)butyramide), DPPG (1,2-dipalmitoyl-*sn*-

glycero-3-phospho-(1'-*rac*-glycerol)), DSPC (1,2-distearoyl-*sn*-glycero-3-phosphocholine), DSPE (1,2-distearoyl-*sn*-glycero-3-phosphoethanolamine), DSPE-PAA-DNA (poly(acrylic acid) (PAA) is coupled to DSPE via carbodiimide-mediated amidation, two amine terminated DNA strands (5'$H_2N$-CCAACCACAAAA, 5' AAAACAACCCCA-$NH_2$) are then attached to the carboxyl groups of PAA, on average there are 3.3 DNA strands per DSPE-PAA molecule), DSPE-PEG-BTC (distearoylphosphatidylethanolamine-polyethylene glycol-benzotriazole carbonate), DSPE-PEG2000 ((1,2-distearoyl-*sn*-glycero-3-phosphoethanolmine-N-methoxy(polyethylene glycol)-2000), DSPE-PEG2000-Biotin (1,2-distearoyl-*sn*-glycero-3-phosphoethanolamine-N-(biotinyl (polyethylene glycol)-2000)), DSPE-PEG3400-Biotin (1,2-distearoyl-*sn*-glycero-3-phosphoethanolamine-N-(biotinyl (polyethylene glycol)-3400)), DSPE-PEG2000-Maleimide (1,2-distearoyl-*sn*-glycero-3-phosphoethanolamine-N-(maleimide (polyethylene glycol)-2000)); DSPE-PEG3400-Maleimide-peptide (distearoylphosphatidylethanolamine-polyethylene glycol (3400)-Maleimide-peptide), DSPE-PEG-PDP (distearoylphosphatidylethanolamine-PEG 2000-pyridyldithio propionate), DSTAP (1,2-distearoyl-3-trimethylammoniumpropane; provides positive charge), HSPC (hydrogenated soy L-α-phosphatidylcholine), ID (identification), IQR (inter quartile range), GPIIb/IIIa (glycoprotein IIb/IIIa receptor on activated platelets), LPS (lipopolysaccharide), mol (mole), mol% (mole %), mAb (monoclonal antibody), MPB (maleimido-4(p-phenylbutyrate)), NA (not available/applicable), ND (not disclosed), NRT (non real-time imaging), NTHU (National Tsing Hua University, Taiwan), pAb (polyclonal antibody), PC (phosphocholine), PEG (polyethylene glycol), PEG4000 (polyethylene glycol-4000), PEG40-Stearate (mono-stearate poly(ethylene)glycol-40), Q (quantitative imaging), RT (real-time imaging), RT(NS) (real-time non bubble specific mode imaging), scFvs (single-chain antibody), SMU (Southern Medical University, Guangzhou, China), SQ (semi-quantitative imaging), TACS (thrombin aptamer crosslinking sequence: a complementary DNA strand GGTTGGTGTGGTTGGTGTTTTTTTTTGTGGTTGGTGTGGTTGG, it cross-links DNA (of DSPE-PAA-DNA) on the bubble surface, but subsequent interaction with thrombin results in its displacement from the DNA cross-links, making the bubble shell less rigid, and hence more echogenic), Thy1 (thymocyte differentiation antigen 1 - a specific biomarker of pancreatic ductal adenocarcinoma neovasculature), TMMU (Third Military Medical University, Chongqing, China), UC (University of Colorado, USA), UCA Davis (University of California, Davis, USA), UCA La Jolla (University of California, La Jolla, USA), UNC (University of North Carolina, USA), UT (University of Toronto, Canada), UVA (University of Virginia, USA), v/v (volume/volume), WKU (Wonkwang University, Iksan, Jeonbuk, South Korea), XJU (Xi'an Jiaotong University, Xi'an Shaanxi, China), YSPSL (also known as rPSGL-Ig (Y's Therapeutics Inc), a dimeric fusion protein consisting of recombinant human P-selectin glycoprotein ligand-1 protein (rPSGL) fused to a fragment crystallizable (Fc) domain of human immunoglobulin G1, produced using Chinese Hamster Ovary cells). **Keys:** *On tumor derived endothelial cells. †Superparamagnetic microbeads coated (via streptavidin) phospholipid bubbles. ‡By microscopy, which tends to give larger size and smaller concentration measurements compared with electrozone sensing. §BCNU is attached to the phospholipid shell by electrostatic and hydrophobic interactions. BCNU encapsulated in the bubbles = 1.02±0.37mg/mL (bubble concentration = 12.29±0.25 x$10^9$ bubbles/mL). | |Avidin on the bubbles target biotinylated anti-glycoprotein IIb/IIIa receptor mAb administered *iv* 30min prior to *iv* administration of the bubbles. # Free unmodified YSPSL has completed Phase 2a clinical trial in renal transplant patients [2]. Modified YSPSL (biotinylated [3, 4], or thiolated using 2-iminothiolane (Traut's reagent) [5, 6] /an undisclosed method [7]) were used for bubble conjugation. **In order for the targeting bubbles (confined to the intravascular space) to target MMP2, microvascular permeability was transiently increased by triggered myocardial contrast echocardiography. ††Disuccinimidyl glutarate linking amine on targeting peptide with amine on DSPE-PEG2000, producing (DSPE-PEG2000)-disuccinimidyl glutarate-(peptide). This conjugation strategy introduces glutaric acid linker between the phospholipid-PEG and peptide. ‡‡Microbubble shell outer surface contains a network of single-stranded oligonucleotides (DNA from DSPE-PAA-DNA) which are cross-linked by TACS (a complementary DNA strand). This increases the rigidity of the bubble shell, reducing its echogenicity. In the presence of clinically-relevant, elevated amounts of thrombin, TACS preferentially binds to free thrombin, thus displaced from the DNA cross-links. This decreases the rigidity of the bubble shell, increasing its echogenicity. §§Quantitative or semi-quantitative imaging is achieved when acoustic quantification of the targeted molecule correlates with that using an independent (non-acoustic) quantification method which is quantitative or semi-

quantitative in nature, respectively. ##Full methodology for bubble preparation disclosed.

# Supplemental References


1. Klibanov AL, Gu H, Wojdyla JK, Wible JH, Jr., Kim DH, Needham D, et al. Attachment of ligands to gas-filled microbubbles via PEG spacer and lipid residues anchored at the interface. Proceed Int'l Symp Control Rel Bioact Mater. 1999;26:230.
2. Gaber AO, Mulgaonkar S, Kahan BD, Woodle ES, Alloway R, Bajjoka I, et al. YSPSL (rPSGL-Ig) for improvement of early renal allograft function: a double-blind, placebo-controlled, multi-center Phase IIa study. Clinical transplantation. 2011;25(4):523-33. doi: 10.1111/j.1399-0012.2010.01295.x. PubMed PMID: 20573162.
3. Davidson BP, Kaufmann BA, Belcik JT, Xie A, Qi Y, Lindner JR. Detection of antecedent myocardial ischemia with multiselectin molecular imaging. J Am Coll Cardiol. 2012;60(17):1690-7. doi: 10.1016/j.jacc.2012.07.027. PubMed PMID: 23021335.
4. Bettinger T, Bussat P, Tardy I, Pochon S, Hyvelin JM, Emmel P, et al. Ultrasound molecular imaging contrast agent binding to both E- and P-selectin in different species. Investigative radiology. 2012;47(9):516-23. PubMed PMID: 22814589.
5. Wang H, Machtaler S, Bettinger T, Lutz AM, Luong R, Bussat P, et al. Molecular imaging of inflammation in inflammatory bowel disease with a clinically translatable dual-selectin-targeted US contrast agent: comparison with FDG PET/CT in a mouse model. Radiology. 2013;267(3):818-29. doi: 10.1148/radiol.13122509. PubMed PMID: 23371306; PubMed Central PMCID: PMC3662899.
6. Hyvelin JM, Tardy I, Bettinger T, von Wronski M, Costa M, Emmel P, et al. Ultrasound molecular imaging of transient acute myocardial ischemia with a clinically translatable p- and e-selectin targeted contrast agent: correlation with the expression of selectins. Investigative radiology. 2014;49(4):224-35. doi: 10.1097/RLI.0000000000000018. PubMed PMID: 24442162.
7. Davidson BP, Chadderdon SM, Belcik JT, Gupta S, Lindner JR. Ischemic memory imaging in nonhuman primates with echocardiographic molecular imaging of selectin expression. J Am

Soc Echocardiogr. 2014;27(7):786-93 e2. doi: 10.1016/j.echo.2014.03.013. PubMed PMID: 24774222; PubMed Central PMCID: PMC4065817.
